# Inflammation PET and plasma neurofilament light predict survival in people with progressive supranuclear palsy

**DOI:** 10.1093/braincomms/fcaf467

**Published:** 2025-11-27

**Authors:** Noah L Shapiro, Peter Simon Jones, Elijah Mak, Kamen A Tsvetanov, Julia Goddard, Davi S Vontobel, Robert Durcan, Leonidas Chouliaras, Tim Fryer, Young T Hong, Franklin Aigbirhio, Amanda Heslegrave, Nicolai Franzmeier, Matthias Brendel, Henrik Zetterberg, John T O’Brien, James B Rowe, Maura Malpetti

**Affiliations:** Department of Clinical Neurosciences, University of Cambridge, Cambridge, CB2 0SZ, UK; UK Dementia Research Institute at the University of Cambridge, Cambridge, CB2 0AH, UK; Department of Clinical Neurosciences, University of Cambridge, Cambridge, CB2 0SZ, UK; Department of Psychiatry, School of Clinical Medicine, Addenbrooke’s Hospital, University of Cambridge, Cambridge, CB2 0SZ, UK; Department of Clinical Neurosciences, University of Cambridge, Cambridge, CB2 0SZ, UK; Department of Psychology, University of Cambridge, Cambridge, CB2 3EB, UK; Department of Clinical Neurosciences, University of Cambridge, Cambridge, CB2 0SZ, UK; UK Dementia Research Institute at the University of Cambridge, Cambridge, CB2 0AH, UK; Department of Clinical Neurosciences, University of Cambridge, Cambridge, CB2 0SZ, UK; UK Dementia Research Institute at the University of Cambridge, Cambridge, CB2 0AH, UK; Department of Clinical Neurosciences, University of Cambridge, Cambridge, CB2 0SZ, UK; Department of Psychiatry, School of Clinical Medicine, Addenbrooke’s Hospital, University of Cambridge, Cambridge, CB2 0SZ, UK; Department of Clinical Neurosciences, University of Cambridge, Cambridge, CB2 0SZ, UK; Wolfson Brain Imaging Centre, University of Cambridge, Cambridge, CB2 0QQ, UK; Department of Clinical Neurosciences, University of Cambridge, Cambridge, CB2 0SZ, UK; Wolfson Brain Imaging Centre, University of Cambridge, Cambridge, CB2 0QQ, UK; Department of Clinical Neurosciences, University of Cambridge, Cambridge, CB2 0SZ, UK; Wolfson Brain Imaging Centre, University of Cambridge, Cambridge, CB2 0QQ, UK; UK Dementia Research Institute and Department of Neurodegenerative Disease at UCL, London, NW1 3BT, UK; Munich Cluster for Systems Neurology (SyNergy), Munich, 81377, Germany; Institute for Stroke and Dementia Research (iSD), University Hospital, Ludwig-Maximilians-Universität, Munich, 81377, Germany; Department of Psychiatry and Neurochemistry, Institute of Neuroscience and Physiology, Sahlgrenska Academy at University of Gothenburg, Mölndal, 431 39, Sweden; Munich Cluster for Systems Neurology (SyNergy), Munich, 81377, Germany; Institute for Stroke and Dementia Research (iSD), University Hospital, Ludwig-Maximilians-Universität, Munich, 81377, Germany; Bavarian Cancer Research Center (BZKF), Partner Site Munich, Munich, 80539, Germany; German Cancer Consortium (DKTK), Partner Site Munich, a partnership between German Cancer Research Center (DKFZ) and LMU Munich, Munich, 80366, Germany; Department of Nuclear Medicine, LMU University Hospital, LMU Munich, Munich 81377, Germany; German Center for Neurodegenerative Diseases (DZNE) Munich, Munich, 81377, Germany; UK Dementia Research Institute and Department of Neurodegenerative Disease at UCL, London, NW1 3BT, UK; Department of Psychiatry and Neurochemistry, Institute of Neuroscience and Physiology, Sahlgrenska Academy at University of Gothenburg, Mölndal, 431 39, Sweden; Department of Neurodegenerative Disease, Clinical Neurochemistry Laboratory, Sahlgrenska University Hospital, Mölndal, 405 30, Sweden; Wisconsin Alzheimer’s Disease Research Center, University of Wisconsin School of Medicine and Public Health, University of Wisconsin-Madison, Madison, WI 53792, USA; Hong Kong Center for Neurodegenerative Diseases, Clear Water Bay, Hong Kong, China; Department of Psychiatry, School of Clinical Medicine, Addenbrooke’s Hospital, University of Cambridge, Cambridge, CB2 0SZ, UK; Department of Clinical Neurosciences, University of Cambridge, Cambridge, CB2 0SZ, UK; Cambridge University Hospitals NHS Foundation Trust, Cambridge, CB2 0QQ, UK; Medical Research Council Cognition and Brain Sciences Unit, University of Cambridge, Cambridge, CB2 7EF, UK; Department of Clinical Neurosciences, University of Cambridge, Cambridge, CB2 0SZ, UK; UK Dementia Research Institute at the University of Cambridge, Cambridge, CB2 0AH, UK; Cambridge University Hospitals NHS Foundation Trust, Cambridge, CB2 0QQ, UK

**Keywords:** neuroinflammation, survival, progressive supranuclear palsy, PET NfL

## Abstract

Progressive supranuclear palsy (PSP) is a primary tauopathy characterized by atrophy and neuroinflammation of the brainstem, the basal ganglia and, to a lesser degree, the cortex. This study investigates the association of regional atrophy (structural MRI), neuroinflammation ([^11^C]-PK11195 PET), peripheral markers of neurodegeneration [plasma neurofilament light chain (NfL)] and clinical severity [PSP rating scale (PSPRS)] with survival in people with PSP. Fifty-nine people with PSP underwent longitudinal structural MRI, surviving on average 3.2 years from the first scan (MRI cohort). Sixteen participants (PET cohort) within this cohort underwent cross-sectional [^11^C]-PK11195 PET and blood sampling for plasma NfL. We applied modality-specific principal component analyses on imaging data and ran partial correlations, multivariate regressions and Bayesian models to evaluate the association between survival and imaging patterns, clinical severity and plasma NfL. In the PET cohort, higher levels of localized inflammation in subcortical regions [rho = −0.49, *P* = 0.02, Bayes factor (BF) = 8.07] and plasma NfL (rho = −0.57, *P* = 0.01, BF = 4.63) were associated with shorter survival, while PSPRS scores were not significant predictors of survival. Subcortical atrophy was associated with shorter survival in the larger cohort (*r* = −0.38, *P* = 0.001; *β* = −0.66, *P* = 0.001). Spearman’s correlations, multivariate regressions and Bayesian models converged to the same results. Regional subcortical atrophy is a robust biomarker associated with survival in people with PSP that can be utilized in large-scale clinical trials. Translocator protein (TSPO) PET and plasma NfL offer promising complementary markers for smaller-scale trials, where they may prove more sensitive than clinical scores or structural MRI alone. By linking neuroinflammation to survival, our results also highlight immunotherapy as a promising avenue for disease-modifying treatment in PSP.

## Teaser

Shapiro *et al.* report that inflammation PET signals in subcortical regions and plasma neurofilament light levels are complementary prognostic biomarkers in progressive supranuclear palsy, demonstrating greater sensitivity than other measures. They link neuroinflammation to survival, further highlighting its central role in disease progression and potential as a target for disease-modifying therapies.

## Graphical Abstract

**Graphical Abstract:**
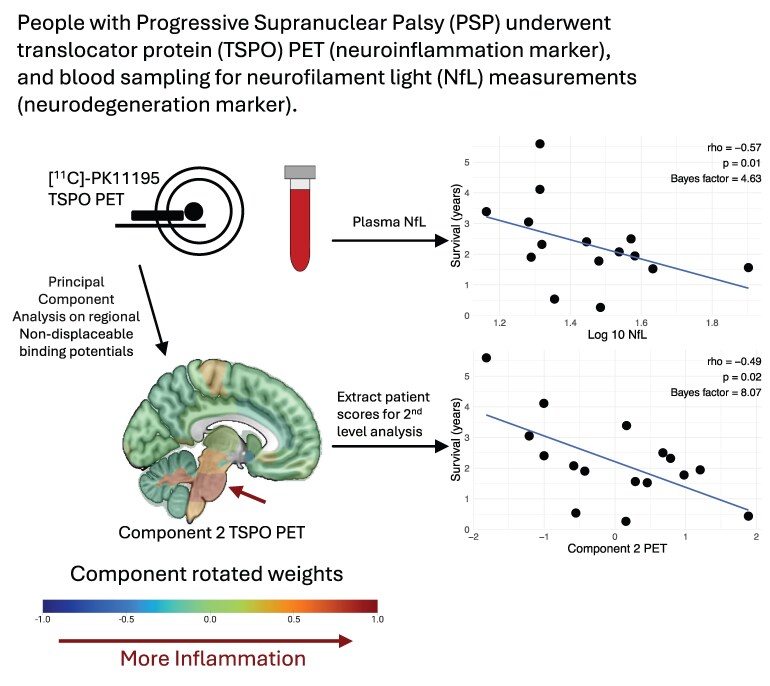

## Introduction

Progressive supranuclear palsy (PSP) is a primary tauopathy, presenting with postural instability, supranuclear palsy, akinesia and cognitive change.^1^ The early diagnosis of PSP can be challenging, although clinicopathological correlations are high.^1^ Biomarkers of PSP have been sought to improve diagnosis, prognostication and pathophysiology characterization of PSP. For example, progressive atrophy in the brainstem, the basal ganglia and later the cortex is identifiable by magnetic resonance imaging (MRI).^2,3^ Four-repeat tau pathology and synaptic loss associated with PSP are identifiable by positron emission tomography (PET) with second-generation tau ligands and SV2 ligands. The neurofilament light chain (NfL) has emerged as a marker of ‘non-specific’ axonal injury and neurodegeneration,^4^ reflecting its predominant expression in axons.^5^ In people with PSP, NfL levels in plasma and cerebrospinal fluid (CSF) are substantially increased.^6,7^ Longitudinal assessment of plasma NfL has been implemented in clinical trials in people with PSP^8^ showing associations with clinical progression.^6^

Beyond tau pathology and neurodegeneration, neuroinflammation with microglial activation and astrogliosis is a key contributor to PSP pathogenesis. Human post-mortem studies showed that activated microglia co-localize with neuronal fibrillary inclusions in subcortical and cortical regions.^9,10^ PET has enabled the localization and quantification of neuroinflammation in PSP. The most commonly used PET ligands aiming to characterize neuroinflammation target the 18 kDa translocator protein (TSPO), which is overexpressed in the outer mitochondrial membrane of microglia.^11^ Several TSPO radioligands have been implemented in neurodegenerative diseases,^12^ including the first-generation [^11^C]-PK11195.^13^ In PSP, [^11^C]-PK11195 PET shows increased signals in subcortical regions,^14,15^ co-localizing with tau pathology,^16^ and associates with clinical decline.^17^

Most studies utilize the PSP rating scale (PSPRS) to track disease progression in people with PSP. The PSPRS is a clinically valid and widely used test to characterize disease severity in people with PSP.^18^ However, some items on the PSPRS are not highly sensitive to change, are affected by ceiling effects^19^ and need to be complemented by other functional measures of activities of daily living, dysphagia and cognition to enable a complete assessment of clinical decline. While PSPRS provides a more immediate and quantifiable measure of clinical deterioration, in retrospective studies, survival offers a broader and more objective assessment of disease impact over time.

In this study, our primary outcomes are the association between localized microglial-mediated neuroinflammation (measured by [^11^C]-PK11195) and plasma NfL levels with survival in a small sample size of well-phenotyped people with PSP-Richardson’s syndrome (PSP-RS). Our secondary outcome compares the associations of [^11^C]-PK11195 PET, plasma NfL, structural MRI or PSPRS scores with survival. Our tertiary outcomes replicate previous findings to confirm whether atrophy patterns in subcortical regions and PSPRS scores are associated with survival in a larger and clinically heterogeneous cohort of participants with PSP.

## Materials and methods

### Participants

To address the primary and secondary outcomes, we selected data from 16 deceased people with PSP-RS that underwent [^11^C]-PK11195 PET and MRI, as part of the Neuroimaging of Inflammation in Memory and Related Other Disorders (NIMROD) study.^20^ In addition, PSPRS scores and plasma samples were collected close to the PET scan for quantification of Aβ40, Aβ42, glial fibrillary acidic protein (GFAP), NfL and p-tau181 levels. On average, TSPO PET scans were obtained within ∼4 months from MRI (median interval: 3.46 months), PSPRS assessment (3.45) and blood sampling (0).

We refer to these *N* = 16 participants as the ‘PET cohort’. To conduct the analysis that investigated the association between plasma NfL and survival, we had to exclude one participant who had a PET scan but no plasma samples available.

To address the tertiary outcomes, we selected data from *N* = 59 deceased participants (which includes the *N* = 16 participants from the PET cohort) in long-term observational studies that comprised diverse PSP clinical syndromes, including PSP-RS (78.2%), PSP with predominant frontal presentation (16.4%), PSP with progressive gait freezing (3.6%) and PSP with predominant parkinsonism (1.7%). Participants with PSP underwent clinical assessment, including PSPRS and baseline and follow-up MRI scans (see Supplementary Fig. 1 for the distribution of MRI visits across years). We refer to these *N* = 59 participants as the ‘MRI cohort’.

For both cohorts, participants all met clinical diagnostic Movement Disorder Society-PSP 2017 criteria^1^ under the care of National Health Service clinics affiliated with the Cambridge University Centre for Parkinson-Plus. Trained neurologists administered the PSPRS, who may have seen patients and related assessment as part of their NHS clinical appointments, but were not aware of research data results at the time of the PSPRS assessment. By the time of this study, *N* = 13 had donated their brain to the Cambridge Brain Bank, with a neuropathological diagnostic confirmation of PSP in all of them.

Participants with mental capacity gave their written informed consent to take part in the study. For those who lacked capacity, their participation followed the personal consultee process in accordance with UK law. The research protocols were approved by the National Research Ethics Service’s East of England Cambridge Central Committee and the UK Administration of Radioactive Substances Advisory Committee.

### Imaging parameters and processing

Sixteen patients underwent [^11^C]-PK11195 PET, using dynamic imaging for 75 min, with GE Advance and GE Discovery 690 PET/CT scanners (GE Healthcare, Waukesha, USA). All PET scans were performed using the R-enantiomer of [^11^C]-PK11195. For consistency, we refer to it throughout the manuscript as [^11^C]-PK11195. In this sub-group of patients (PET cohort), the interval between [^11^C]-PK11195 and cross-sectional MRI scans had mean and standard deviation (SD) of 3.8 ± 3.1 months.

The MRI imaging protocols have been described previously.^20,21^ In brief, all 59 participants (MRI cohort) underwent T1-weighted (T1w) scans resampled to 1 mm^3^ voxel sizes on a 3T MRI machine on Prisma Fit (32.2%), TrioTim (44.1%), Skyra Fit (11.9%), Verio (5.1%) and Signa PET MR (6.8%) (Siemens, Erlangen, Germany, GE Medical Systems, Milwaukee, WI, USA). For further information on the scanning parameters, refer to Supplementary Table 11. All participants underwent one MRI scan at baseline, and *n* = 55 had further MRI scans at follow-up visits (up to seven visits; <6.07 years from baseline); for a detailed overview on the visit distributions, refer to Supplementary Fig. 1. For a detailed description of the quality checks and processing of MRI and PET, refer to the Supplementary material.

For the MRI cohort, the first MRI obtained is referred to as the MRI at baseline, whereas within the PET cohort, the MRI obtained closest to the PET is referred to as the cross-sectional MRI.

### Blood sample collection and processing

Blood samples were obtained by venepuncture and collected in ethylenediaminetetraacetic acid tubes,^22^ centrifuged to isolate plasma, then aliquoted and stored at −70°C. Plasma assays were conducted at the UK Dementia Research Institute biomarker laboratory. For a detailed description of blood processing, refer to the Supplementary material. To normalize the single biomarker values, they were log_10_-transformed before statistical analyses.

### Statistical analyses

Statistical analyses were performed in RStudio (version 4.3.2).

For the MRI cohort, survival was defined as the time in years between the MRI scan at baseline and death. For the PET cohort, survival was defined as the time interval between the TSPO PET scan and death.

For time intervals between modalities (MRI, PET, PSPRS and blood draw), we fitted a one-way analysis of variance (ANOVA) to quantify whether the mean difference between modalities was significantly different. For the remaining variables, we calculated the mean and SD across the PSP cohort (Table 1).

**Table 1:** Demographic, clinical and biomarker details of participants with PSP

	MRI full cohort	PET sub-cohort
*N* patients	59	16
Sex (F/M)	28/31	7/9
Age at MRI (mean ± SD)	70 ± 7	69 ± 6
Years of education (mean ± SD)	13 ± 3	12 ± 2
Baseline PSPRS score	27 ± 10	42 ± 14
Time from onset to MRI (mean ± SD)	3.7 ± 2.2	5 ± 1.5
Definite PSP at post-mortem	13/13	8/8
NfL pg/mL (median, q1–q2)	-	27.9 (20.60–35.85)
GFAP pg/mL (median, q1–q2)	-	116.39 (107.31–167.90)
p-tau181 pg/mL (median, q1–q2)	-	1.92 (1.70–2.46)

First, a linear mixed effect model was applied to the longitudinal regional brain volumes across *N* = 59 to estimate the annualized rate of brain volume changes (MRI slopes) after testing for the model assumptions. We allowed the model to estimate subject-specific intercepts and slopes (annual rate of change) where the regional brain volume was predicted by time (in years). Then, subject-specific slopes were extracted and included in further analyses.

Second, for each modality in the MRI cohort (*N* = 59; MRI at baseline and MRI slopes) and in the PET cohort (*N* = 16; cross-sectional MRI and [^11^C]-PK11195 PET), a principal component analysis (PCA) was performed separately to reduce the dimensionality of the four modality-specific datasets. To enhance the interpretability of the resulting components, we flipped the variable- and participant-specific loadings onto the components to fit the neurobiological interpretations: lower values on MRI components reflect volume loss, while higher values on TSPO PET components reflect higher inflammation. Bartlett’s test on the four modality-specific datasets confirmed that the variables could be meaningfully reduced in components. We retain components that had eigenvalues > 1 (Kaiser criterion) and cumulative explained variance ≥ 80%. For each modality, we applied a varimax rotation on all retained components and extracted subject scores for second-level analyses.

Next, we implemented association analyses. As for *a priori* hypothesis, we focused on the modality-specific component loaded onto PSP core regions, including the brainstem, cerebellum and basal ganglia, and we investigated one-tailed correlation analyses. Specifically, we tested whether proxies of neurodegeneration (lower MRI volumes and higher plasma NfL levels), neuroinflammation (higher PET signals) or clinical severity (higher PSPRS scores) were associated with shorter survival.

To address the primary and secondary outcomes in the PET cohort, we applied partial Spearman’s correlations to test the association of survival (time from PET scan to death) with (i) subcortical atrophy (volumes on cross-sectional MRI), (ii) subcortical inflammation, as quantified by [^11^C]-PK11195 PET, (iii) plasma NfL levels and (iv) clinical severity (PSPRS score). Each correlation analysis included disease duration (time from onset to PET) as a covariate. Given the small sample size of the cohort, we applied both Frequentist and Bayesian Spearman’s correlations (https://osf.io/gny35/), allowing more robust statistical interpretation by Bayes factors (BF). Next, we ran linear models on PSP core components and plasma NfL levels to associate with survival, adjusting for sex, age, years of education and disease duration. Beyond the hypothesis-driven analyses, we ran explorative correlation analyses with the remaining components and survival, adjusting for disease duration. Finally, Fisher’s *Z* transformation analysis^23^ included modality-specific significant rho correlation coefficients to compare associations between modalities and survival. A *P*-value > 0.05 suggests neither modality is more strongly associated with survival.

To address the tertiary outcomes, we tested the prognostic value of baseline MRI and annual rates of change in regional volumes. Across *n* = 59 (MRI cohort) patients with baseline and longitudinal MRI scans, as assumptions for parametric tests were met, we applied Pearson’s correlations between modality-specific components and survival, correcting for disease duration. We further tested the prognostic value of the PSPRS on survival. Each correlation was complemented by linear models, including survival as the outcome variable, the modality-specific PSP core component as a main predictor (or the PSPRS) and sex, age, years of education and disease duration as covariates. Beyond the hypothesis-driven analyses, we ran explorative correlation analyses with the remaining imaging components and survival, adjusting for disease duration.

Lastly, we fitted Kaplan–Meier survival curves with the MRI component at baseline, MRI slope component and PSPRS scores separately as single predictors in the MRI cohort. We stratified each predictor in quantiles (q), defined as ‘high’ (*x* > q3), ‘medium’ (q1 < *x* < q3) and ‘low’(*x* < q1) (Supplementary Fig. 3A–C). For the PET cohort, we also fitted Kaplan–Meier curves with [^11^C]-PK11195, plasma NfL and MRI cross-sectional component scores. As the sample size was smaller than that of the MRI cohort, we stratified the predictors into ‘high’ and ‘low’ scores, using the median as cut-offs (Supplementary Fig. 3D–F).

For all correlation analyses, we set the alpha at 0.05 to reject or not reject the null hypothesis and estimated 95% confidence intervals (95% CI) of the correlation coefficient. We set a BF ≥ 3 to quantify evidence supporting the alternative hypothesis under the Lee and Wagenmakers^24^ criteria. In addition, we also highlight effects with a BF ≥ 2 that support ‘anecdotal’ evidence of the alternative hypothesis, indicating preliminary findings that warrant further investigations.

## Results

Table 1 provides demographic and biomarker summaries and clinical details of patients in each cohort. See Supplementary Fig. 2 for the distribution of survival times from baseline MRI to death in years. For the PET cohort, a one-way ANOVA was applied to the time between PET and each of the other predictors considered. The ANOVA indicated that the time lapse between modalities was not statistically significant [*F*(2,44) = 1.23, *P* = 0.3].

### PET cohort

#### Subcortical inflammation but not cortical inflammation is associated with survival

**Figure 1:**
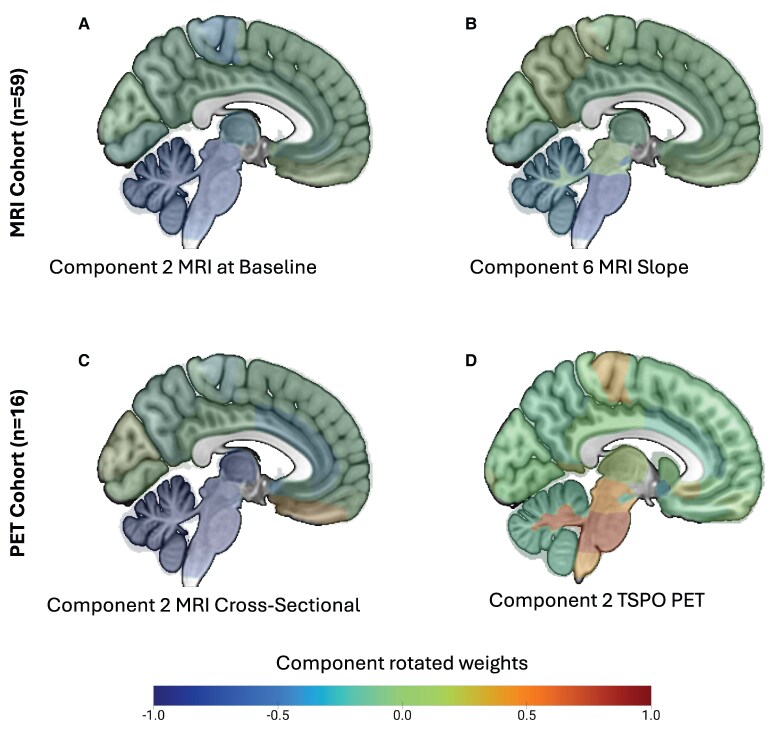
**Components reflecting patterns of atrophy and neuroinflammation in PSP core regions**. PCA of the MRI cohort identified subcortical components of the baseline MRI volumes (**A**) and volumetric slopes (annual rate of change) (**B**). PCA of the PET cohort identified subcortical components in the regional MRI volumes (**C**) (on the MRI scan closest to PET) and in regional neuroinflammation (**D**) indexed by [^11^C]-PK11195 (TSPO PET). See Supplementary Tables 7–10 for the other significant components. The colour bar indicates regional contributions (correlations) to each component: in MRI components, regions at the lower end of the scale (left, blue) represent the most atrophic areas, while in the PET component, regions at the upper end of the scale (right, red) represent highly inflamed regions. Brain images were rendered using MRIcroGL (Rorden & Brett, 2000).

**Figure 2:**
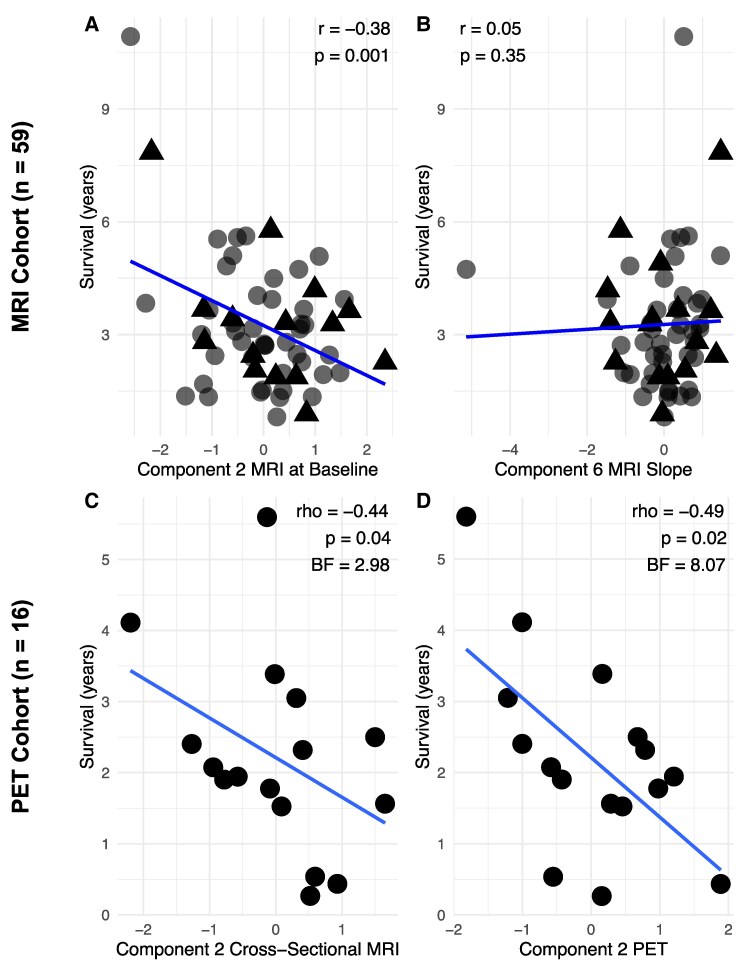
**Subcortical atrophy and neuroinflammation are associated with shorter survival**. Scatter plots for the MRI cohort show partial Pearson’s correlations between the hypothesis-driven subcortical and brainstem components (Components 2 and 6) of MRI at baseline (**A**) and for MRI slope (**B**) with survival, controlled for disease duration. The line indicates the best fit. The triangles represent individual participants, where the red dots indicate participants who are also part of the PET cohort. Scatter plots for the PET cohort show partial Spearman’s correlations supplemented by Bayesian Spearman’s correlations between the hypothesis-driven subcortical and brainstem components of MRI (**C**) and of TSPO PET (**D**) with survival, controlled for disease duration.

The PET-specific PCA identified four components from [^11^C]-PK11195 regional values (cumulative explained variance 82%). The second component captured subcortical inflammation and was loaded onto PSP core subcortical regions (Fig. 1D). Notably, the first component captured cortical inflammation. For regional loadings onto the remaining components, see Supplementary Table 7. Spearman’s partial correlation between survival and the PET PSP core component (Component 2) identified a significant negative association [rho = −0.49, 95% CI (−0.79, 0.007), *P* = 0.02, BF = 8.07; Fig. 2D]. In the linear model the component demonstrated a significant negative association (*β* = −0.96, standard error (SE) = 0.39, *t* = −2.45, *P* = 0.03, *R*² = 0.40). See Supplementary Table 4 for explorative correlations with the other components, which did not identify any significant association with survival.

#### Plasma NfL is associated with survival

**Figure 3:**
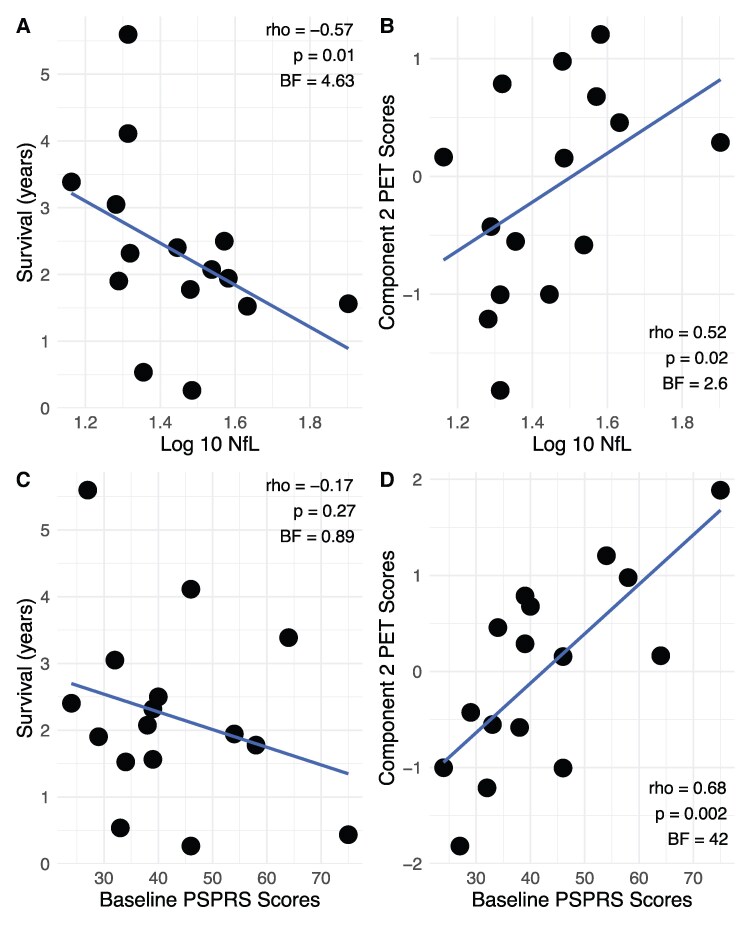
**Plasma NfL levels, but not PSPRS scores, correlate with survival in the PET cohort**. All scatter plots show partial Spearman’s correlation supplemented by Bayesian Spearman’s correlations. (**A**) Scatter plot shows the association between the log_10_-transformed NfL levels and survival, controlled for disease duration. (**C**) Scatter plot shows the association between baseline PSPRS scores and survival, controlled for disease duration. (**B**, **D**) Scatter plots represent, respectively, associations of log_10_-transformed NfL levels and of PSPRS scores with subcortical inflammation. The dots are participants from the PET cohort, and the line represents the line of best fit.

Spearman’s partial correlation between survival and plasma NfL identified a significant negative association [rho = −0.57, 95% CI (−0.83, −0.08), *P* = 0.01, BF = 4.63; Fig. 3A]. In the linear model (*R*² = 0.32), plasma NfL levels were not statistically significantly associated with survival (*β* = −4.80, SE = 2.43, *t* = −1.97, *P* = 0.08). See Supplementary Table 6 for exploratory correlations with other plasma biomarkers. Notably, plasma NfL levels significantly correlated with PET PSP core subcortical Component 2 [rho = 0.52, 95% CI (0.01, 0.81), *P* = 0.02, BF = 2.6; Fig. 3B] but not with the PET thalamocortical component [Component 1; rho = −0.09, 95% CI (−0.57, 0.44), *P* = 0.6, BF = 0.24].

Including an interaction term between plasma NfL levels and subcortical inflammation did not identify significant interaction effects on survival (NfL*Component 2: *β* = 4.97, SE = 4.26, *t* = 1.16, *P* = 0.28).

To examine whether the association of PET with survival does not reflect structural loss on MRI, we included both PET and MRI subcortical components into a linear model with survival as the outcome variable. The PET component was statistically significantly associated with survival (*β* = −0.84, SE = 0.41, *t* = −2.03, *P* = 0.036), whereas the MRI component was not (*β* = −0.36, SE = 0.40, *t* = −0.903, *P* = 0.195).

#### The clinical PSPRS score is not associated with survival

Spearman’s partial correlation between survival and baseline PSPRS scores identified no statistically significant association [rho = −0.17, 95% CI (−0.61, 0.33), *P* = 0.27, BF = 0.89; Fig. 3C]. In the linear model, baseline PSPRS scores were not statistically significantly associated with survival (*β* = −0.02, SE = 0.03, *t* = −0.625, *P* = 0.5, *R*^2^ = 0.09). Notably, the PET PSP core component (subcortical Component 2) significantly correlated with baseline PSPRS [rho = 0.68, 95% CI (0.27, 0.87), *P* = 0.001, BF = 42; Fig. 3D]. Further baseline PSPRS does not correlate with plasma NfL levels [rho = 0.14, 95% CI (−0.40, 0.68), *P* = 0.30, BF = 0.32].

#### Structural MRI is associated with survival in the PET cohort

In the cohort of patients with PET (*n* = 16), the PCA on cross-sectional MRI identified six components (cumulative explained variance 81%). Component 2 captures atrophy in PSP core regions (Fig. 1C). For regional loadings onto the remaining components, see Supplementary Table 8. Spearman’s partial correlation between survival rates and the cross-sectional MRI PSP core component (Component 2) identified a significant negative association [rho = −0.44, 95% CI (−0.76, 0.07), *P* = 0.04, one-tailed, BF = 2.98] (Fig. 2C). In the linear model, the component was not statistically significantly associated with survival (*β* = −0.61, SE = 0.44, *t* = −1.39, *P* = 0.19, *R*² = 0.20). See Supplementary Table 5 for exploratory correlations with the other components.

#### Comparisons of MRI, PET and plasma NfL associations with survival

Fisher’s *Z*-test compared the rho correlation coefficients of PET–survival (rho_PET_ = −0.49, *n*_PET_ = 16) and NfL–survival (rho_NfL_ = −0.57, *n*_NfL_ = 15) associations, suggesting no significant differences (*z* = 0.27, *P* = 0.78). Fisher’s *Z*-test also compared MRI–survival (rho_MRI_ = −0.46, *n*_MRI_ = 16) and PET–survival (rho_PET_ = −0.49, *n*_PET_ = 16) associations, showing no statistically significant differences (*z* = −0.09, *P* = 0.92). Lastly, Fisher’s *Z*-test compared the rho correlation coefficients of MRI–survival (rho_MRI_ = −0.46, *n*_MRI_ = 16) and NfL–survival (rho_NfL_ = −0.57, *n*_NfL_ = 15) associations, showing no statistically significant differences (*z* = 0.37, *P* = 0.70).

### MRI cohort

#### Structural MRI is associated with survival in the MRI cohort

Consistent with the established literature, we found that most ROIs showed significant volume loss over time (Supplementary  Table 1 and Supplementary Fig. 4). The PCA on regional MRI volumes at baseline identified 10 significant components (cumulative explained variance 81%). Component 2 captures atrophy in PSP core regions (Fig. 1A). For regional loadings onto the remaining components, see Supplementary Table 10. The PCA on regional annual rates of change (slopes) also identified 10 significant components (cumulative explained variance of 80%). Component 6 captures the rate of brain volume loss in PSP core regions (Fig. 1B). For regional loadings onto the remaining components, see Supplementary Table 9.

The Pearson partial correlation between survival and the baseline MRI PSP core component (Component 2) showed a significant negative association [*r* = −0.38, 95% CI (−0.57, −0.13), *P* = 0.001; Fig. 2A]. In the linear model, the component showed a significant negative associative effect (*β* = −0.66, SE = 0.20, *t* = −3.282, *P* = 0.001, *R*^2^ = 0.32) alongside years of education (*β* = −0.17, SE = 0.06, *t* = −2.67, *P* = 0.01). See Supplementary Table 2 for explorative correlations with the other components. The Pearson partial correlation between survival and the MRI slope PSP core component (Component 6) showed no significant negative association [*r* = 0.05, 95% CI (−0.20, 0.30), *P* = 0.35; Fig. 2B]. In the linear model, the component was not statistically significantly associated with survival (*β* = 0.15, SE = 0.21, *t* = 0.73, *P* = 0.46, *R*^2^ = 0.19). See Supplementary Table 3 for exploratory correlations with the other components.

#### The clinical PSPRS score is not associated with survival in the MRI cohort

The Pearson partial correlation between survival and the PSPRS showed a significant negative correlation [*r* = −0.25, 95% CI (−0.47, 0.001), *P* = 0.02]. In the linear model, however, the PSPRS showed no significant effect (*β* = −0.05, SE = 0.02, *t* = −1.978, *P* = 0.05, *R*^2^ = 0.16) on survival where years of education was a better predictor of survival (*β* = −0.17, SE = 0.07, *t* = −2.53, *P* = 0.01).

## Discussion

Our study found that higher subcortical neuroinflammation, quantified by [^11^C]-PK11195, and neurodegeneration, reflected by plasma NfL levels, are both associated with shorter survival in people with PSP. However, significant interaction effects between plasma NfL and subcortical inflammation on survival were not identified, with Frequentist and Bayesian approaches converging to the same conclusion. In the larger cohort, we also replicated previous findings showing that regional atrophy in subcortical regions is associated with survival. Regional subcortical atrophy is a robust prognostic biomarker that can be utilized in large-scale clinical trials, while TSPO PET and plasma NfL may offer complementary and more sensitive information for smaller-scale trials.

Neuroinflammation has been previously described as part of the pathological changes associated with PSP. Specifically, post-mortem and TSPO PET studies showed that neuroinflammation co-localizes and parallels with the progression of tau pathology from subcortical to cortical regions.^16,25,26^ Inflammation in PSP core subcortical regions has also been shown to associate with faster clinical progression in people with PSP, considering longitudinal PSPRS scores, which captures worsening of symptoms.^17^ Our study expanded on the previous evidence, identifying that subcortical localized inflammation is also associated with shorter survival in people with PSP. We used survival instead of longitudinal PSPRS to capture a definitive and objective aspect of prognosis. Considering survival as a clinical outcome, previous studies found peripheral markers of neuroinflammation being clinically relevant and significant prognostic measures in PSP and related conditions. High serum levels of pro-inflammatory cytokines, associated with neuroinflammation, have been linked with shorter survival in patients on the frontotemporal lobar degeneration spectrum.^27^ The association between TSPO PET and inflammatory cytokines allows us to disentangle central inflammatory processes and its role in accelerated disease progression. In PSP, higher numbers of classical monocytes and natural killer cells, associated with lower numbers of TREM2+ cells, were associated with shorter survival.^28^ Furthermore, genetic variations at the leucine-rich repeat kinase 2 (LRRK2) locus are also associated with survival in PSP,^29^ highlighting the mechanistic contribution of immune pathways to PSP progression. LRRK2-lowering therapies have been proposed in clinical trials for other neurogenerative diseases.^30^ However, the link between LRRK2 levels and regional *in vivo* brain inflammation in PSP remains to be elucidated. Future research may clarify the relationships between peripheral markers of inflammation, LRRK2 and brain inflammation and whether patients with PSP might benefit from LRKK2-lowering or cytokine-targeting therapies.

Plasma NfL is a marker of axonal injury and proxy of neurodegeneration, which is commonly used as a biomarker in clinical trials to monitor drug efficacy and disease progression in diverse neurodegenerative disorders.^31-33^ Plasma NfL relates to brain hypometabolism, atrophy over time and clinical progression. In our small cohort, high levels of plasma NfL were associated with shorter survival in people with PSP, aligning with similar findings in larger cohorts.^6,34^ Conversely, GFAP levels were not significantly associated with survival in people with PSP (Supplementary Table 6). Previous research also suggests that GFAP may be a sensitive marker for astrogliosis related to amyloidosis in Alzheimer’s disease^35^ but less useful in non-Alzheimer’s disease pathologies, including PSP.^22^ This, paired with the lack of statistically significant associations between PSPRS scores and survival, suggests that clinical trials using NfL as an outcome of interest may achieve meaningful interpretation of the results with smaller sample sizes, at least at a group level. Interestingly, in our cohort, plasma NfL levels were strongly associated with neuroinflammation in PSP core subcortical regions, indicating that those patients with higher NfL levels have higher localized brain inflammation. On the contrary, plasma NfL levels did not correlate with widespread inflammation in cortical areas. This suggests that NfL levels can inform regional specificity of other PSP core brain changes and provide an accessible predictor of survival at a group level that may be used in future clinical trials. Moreover, plasma NfL may be a better marker than other clinically validated markers for patient stratification in relatively small clinical trials. We suggest that NfL levels should be evaluated in addition to clinical and inflammatory markers in clinical evaluation and prediction of clinical deterioration. To date, no other studies including patients with PSP have investigated the association between NfL and TSPO radiotracers. Thus, further studies are needed to expand and validate our results in other cohorts and with different TSPO tracers.

Beyond plasma NfL, a commonly and clinically used measure of neurodegeneration is structural MRI. Large studies have shown that both cross-sectional and longitudinal MRI volumes in subcortical and brainstem regions are associated with survival in people with PSP.^36,37^ In our MRI cohort (*n* = 59), we replicated this result, showing that subcortical atrophy at baseline is associated with survival in people with PSP. However, in the PET cohort (*n* = 16), we have no statistically significant associations of regional MRI volumetric measures with survival. Similarly, the PSPRS is commonly used in clinics to track clinical severity^18^ and has been previously shown to be predictive of disease progression and survival. In a cohort of 197 people with PSP, higher scores on the PSPRS predicted a shorter life expectancy.^38^ However, in our PET cohort, baseline PSPRS was not significantly associated with survival, although the directionality of the association was as expected. We suggest that the lack of statistically significant associations of structural MRI and PSPRS with survival in the PET cohort may be due to lower sensitivity of these measures, in the small sample size, leading to underpowered analyses. On the contrary, our results suggest that TSPO PET signals in PSP core regions and plasma NfL may be more sensitive and recommendable markers in small-scale clinical trials.

Interestingly, in the MRI cohort (*n* = 59), when baseline MRI was included in the model alongside sex, age, time between onset and MRI scan and years of education, the latter variable was significantly and negatively correlated with survival. Hence, patients with more years of formal education had a shorter life expectancy. This finding results from initial cognitive and brain reserve as described in other neurodegenerative diseases.^39,40^ For example, in Alzheimer’s disease, higher education as a proxy of cognitive reserve is associated with milder clinical symptomology at a comparable level of brain pathology or worse brain pathology at comparable clinical severity. Thus, patients with higher cognitive reserve are more resilient to brain pathology, showing milder symptomatology, but once pathology passes a critical threshold, individuals with former cognitive reserve experience a more rapid decline. As there is limited data on cognitive reserve in PSP, future research in this field could explore composite scores of reserve, such as occupational attainment, leisure and physical activities, bi- or multilingualism or pre-morbid intelligence quotient,^39^ which might reveal mitigating factors throughout the natural history of the disease.

There are several limitations to our study. First, we acknowledge the small sample size of the PET cohort, which does not permit statistically elaborate models. However, our results found consensus across different analytic methods, including Spearman’s correlations and linear regression models. The Bayesian tests confirmed that we had sufficient precision (analogous to power in Frequentist tests) to support the alternative hypotheses with strong evidence (BF > 3) from the 15 patients’ data. The convergence over these statistical approaches mitigates against inadequate power and sample-dependent biases on the estimation of biomarker–survival associations. Although our cohort is larger than or of comparable size of previous PET studies on rare neurodegenerative diseases like PSP, the replication of these findings with larger and multicentre clinical cohorts will represent an important step to establish the generalizability of our results and utility for clinical trials. Second, there is a current debate regarding the specificity of [^11^C]-PK11195 PET in quantifying microglia,^41,42^ over and above astrocytes or vascular endothelium.^43^ However, in people with PSP, antemortem regional [^11^C]-PK11195 binding is positively correlated with post-mortem phagocytic microglia and microglial TSPO, providing direct evidence that [^11^C]-PK11195 reflects microglial-mediated brain inflammation.^11^ Aligning evidence was described by Garland *et al.*,^44^ who found TSPO being overexpressed and related to phagocytotic microglia in post-mortem tissues from patients with Alzheimer’s disease. The [^11^C]PK11195 tracer has limitations, including its relatively low signal-to-noise ratio and low brain penetration, which may affect its sensitivity to visualize and quantify microglia. Nevertheless, this would reduce effect sizes and increase type II errors, rather than leading to false positive findings. Second-generation PET radioligands for TSPO are characterized by higher signal-to-noise ratio than [^11^C]PK11195, but their binding is markedly affected by single nucleotide polymorphisms (rs6971), which cause heterogeneity in PET data and require genetic screening and lead to the exclusion of participants who are low affinity binders. [^11^C]PK11195 binding is less affected by this polymorphism, has well-established methods of kinetic analysis^45^ and has been validated in PSP with PET-to-post-mortem evidence.^11^ Hence, [11C]PK11195 PET was the ligand of choice for this study of PSP. We acknowledge the limited power of the analyses related to the small size of our PET samples. Third, we recruited according to clinical diagnostic criteria, and although clinicopathological correlations of PSP-RS are very high, including 8 of 8 cases in our PET cohort with post-mortem pathology, they are not perfect. Finally, our results may not be generalizable to other cohorts and primary tauopathies. For example, in a cohort of people with amyloid-negative corticobasal syndrome (CBS), a recent TSPO PET using the [^18^F]-GE-180 ligand reported that higher brain inflammation was associated with slower clinical progression, as assessed by longitudinal PSPRS scores.^46^ The discrepancy with our results may be due to several factors, including (i) regional differences, where PSP is characterized by more localized subcortical inflammation while CBS by widespread cortical inflammation; and (ii) differences in disease severity across cohorts, with previous studies in PSP recruiting on average more advanced participants than in CBS cohorts. Further studies are needed to validate previous evidence across cohorts and diseases.

In conclusion, regional subcortical atrophy is a robust biomarker associated with survival in people with PSP that can be utilized in large-scale clinical trials. TSPO PET and plasma NfL may be more suitable biomarkers for small-scale trials than PSPRS or structural MRI. We further provide evidence that immunotherapeutic strategies are promising avenues for slowing disease progression in people with PSP.

## Supplementary Material

fcaf467_Supplementary_Data

## Data Availability

Anonymized data may be shared by request to the senior author from a qualified investigator for non-commercial use. Data sharing may be subject to restrictions according to consent and data protection legislation. R scripts used in data analysis employed standard functions and packages freely available; no new R package or function was generated for this study.

## References

[1] Höglinger GU, Respondek G, Stamelou M, et al Clinical diagnosis of progressive supranuclear palsy: The Movement Disorder Society criteria. Mov Disord. 2017;32(6):853–864. 28467028 10.1002/mds.26987 PMC5516529

[2] Whitwell JL, Höglinger GU, Antonini A, et al Radiological biomarkers for diagnosis in PSP: Where are we and where do we need to be? Mov Disord. 2017;32(7):955–971. 28500751 10.1002/mds.27038 PMC5511762

[3] Picillo M, Abate F, Ponticorvo S, et al Association of MRI measures with disease severity and progression in progressive supranuclear palsy. Front Neurol. 2020;11:603161. 33281738 10.3389/fneur.2020.603161 PMC7688910

[4] Arslan B, Zetterberg H. Neurofilament light chain as neuronal injury marker—What is needed to facilitate implementation in clinical laboratory practice? Clin Chem Lab Med. 2023;61(7):1140–1149. 36880940 10.1515/cclm-2023-0036

[5] Gaetani L, Blennow K, Calabresi P, Di Filippo M, Parnetti L, Zetterberg H. Neurofilament light chain as a biomarker in neurological disorders. J Neurol Neurosurg Psychiatry. 2019;90(8):870–881. 30967444 10.1136/jnnp-2018-320106

[6] Donker Kaat L, Meeter LH, Chiu WZ, et al Serum neurofilament light chain in progressive supranuclear palsy. Parkinsonism Relat Disord. 2018;56:98–101. 29937097 10.1016/j.parkreldis.2018.06.018

[7] Rojas JC, Bang J, Lobach IV, et al CSF neurofilament light chain and phosphorylated tau 181 predict disease progression in PSP. Neurology. 2018;90(4):e273–e281. 29282336 10.1212/WNL.0000000000004859 PMC5798651

[8] Höglinger GU, Litvan I, Mendonca N, et al Safety and efficacy of tilavonemab in progressive supranuclear palsy: A phase 2, randomised, placebo-controlled trial. Lancet Neurol. 2021;20(3):182–192. 33609476 10.1016/S1474-4422(20)30489-0

[9] Hartnell IJ, Woodhouse D, Jasper W, et al Glial reactivity and T cell infiltration in frontotemporal lobar degeneration with tau pathology. Brain. 2024;147(2):590–606. 37703311 10.1093/brain/awad309 PMC10834257

[10] Hartnell IJ, Blum D, Nicoll JAR, Dorothée G, Boche D. Glial cells and adaptive immunity in frontotemporal dementia with tau pathology. Brain. 2021;144(3):724–745. 33527991 10.1093/brain/awaa457

[11] Wijesinghe SS, Rowe JB, Mason HD, et al Post-mortem validation of in vivo TSPO PET as a microglial biomarker. Brain. 2025;148(6):1904–1910. 40036275 10.1093/brain/awaf078 PMC12129730

[12] Werry EL, Bright FM, Piguet O, et al Recent developments in TSPO PET imaging as a biomarker of neuroinflammation in neurodegenerative disorders. IJMS. 2019;20(13):3161. 31261683 10.3390/ijms20133161 PMC6650818

[13] Cagnin A, Brooks DJ, Kennedy AM, et al In-vivo measurement of activated microglia in dementia. The Lancet. 2001;358(9280):461–467. 10.1016/S0140-6736(01)05625-2 11513911

[14] Passamonti L, Rodríguez PV, Hong YT, et al [11c]PK11195 binding in Alzheimer disease and progressive supranuclear palsy. Neurology. 2018;90(22):1989–1996. 10.1212/WNL.0000000000005610 PMC5980519 29703774

[15] Gerhard A, Trender-Gerhard I, Turkheimer F, Quinn NP, Bhatia KP, Brooks DJ. In vivo imaging of microglial activation with [11C](R)-PK11195 PET in progressive supranuclear palsy. Mov Disord. 2006;21(1):89–93. 16108021 10.1002/mds.20668

[16] Malpetti M, Passamonti L, Rittman T, et al Neuroinflammation and tau colocalize in vivo in progressive supranuclear palsy. Ann Neurol. 2020;88(6):1194–1204. 32951237 10.1002/ana.25911 PMC7116392

[17] Malpetti M, Passamonti L, Jones PS, et al Neuroinflammation predicts disease progression in progressive supranuclear palsy. J Neurol Neurosurg Psychiatry. 2021;92(7):769–775. 33731439 10.1136/jnnp-2020-325549 PMC7611006

[18] Golbe LI, Ohman-Strickland PA. A clinical rating scale for progressive supranuclear palsy. Brain. 2007;130(6):1552–1565. 17405767 10.1093/brain/awm032

[19] Grötsch M, Respondek G, Colosimo C, et al A modified progressive supranuclear palsy rating scale. Mov Disord. 2021;36(5):1203–1215. 33513292 10.1002/mds.28470

[20] Bevan-Jones WR, Surendranathan A, Passamonti L, et al Neuroimaging of Inflammation in Memory and Related Other Disorders (NIMROD) study protocol: A deep phenotyping cohort study of the role of brain inflammation in dementia, depression and other neurological illnesses. BMJ Open. 2017;7(1):e013187. 10.1136/bmjopen-2016-013187 PMC5223666 28064175

[21] Street D, Jabbari E, Costantini A, et al Progression of atypical parkinsonian syndromes: PROSPECT-M-UK study implications for clinical trials. Brain. 2023;146(8):3232–3242. 36975168 10.1093/brain/awad105 PMC10393398

[22] Chouliaras L, Thomas A, Malpetti M, et al Differential levels of plasma biomarkers of neurodegeneration in Lewy body dementia, Alzheimer’s disease, frontotemporal dementia and progressive supranuclear palsy. J Neurol Neurosurg Psychiatry. 2022;93(6):651. 35078917 10.1136/jnnp-2021-327788 PMC9148982

[23] Fisher RA . Statistical methods for research workers. 7th edn. Oliver and Boyd; 1938.

[24] Lee MD, Wagenmakers EJ. Bayesian cognitive modeling: A practical course. 1st ed. Cambridge University Press; 2014. doi:10.1017/CBO9781139087759

[25] Malpetti M, Roemer SN, Harris S, et al Neuroinflammation parallels 18F-PI-2620 positron emission tomography patterns in primary 4-repeat tauopathies. Mov Disord. 2024;39(9):1480–1492. 39022835 10.1002/mds.29924

[26] Ishizawa K, Dickson DW. Microglial activation parallels system degeneration in progressive supranuclear palsy and corticobasal degeneration. J Neuropathol Exp Neurol. 2001;60(6):647–657. 11398841 10.1093/jnen/60.6.647

[27] Malpetti M, Swann P, Tsvetanov KA, et al Blood inflammation relates to neuroinflammation and survival in frontotemporal lobar degeneration. Brain. 2024;148(2):493–505. 10.1093/brain/awae269 PMC7617268 39155063

[28] Strauss A, Swann P, Kigar SL, et al Peripheral innate immunophenotype in neurodegenerative disease: Blood-based profiles and links to survival. Mol Psychiatry. 2025;30(5):1985–1994. 39472664 10.1038/s41380-024-02809-w PMC12015116

[29] Jabbari E, Koga S, Valentino RR, et al Genetic determinants of survival in progressive supranuclear palsy: A genome-wide association study. Lancet Neurol. 2021;20(2):107–116. 33341150 10.1016/S1474-4422(20)30394-X PMC7116626

[30] Herbst S, Lewis PA, Morris HR. The emerging role of LRRK2 in tauopathies. Clin Sci. 2022;136(13):1071–1079. 10.1042/CS20220067 PMC9274527 35815712

[31] Ferreira PCL, Ferrari-Souza JP, Tissot C, et al Potential utility of plasma P-tau and neurofilament light chain as surrogate biomarkers for preventive clinical trials. Neurology. 2023;101(1):38–45. 36878697 10.1212/WNL.0000000000207115 PMC10351303

[32] Simrén J, Andreasson U, Gobom J, et al Establishment of reference values for plasma neurofilament light based on healthy individuals aged 5–90 years. Brain Commun. 2022;4(4):fcac174. 35865350 10.1093/braincomms/fcac174 PMC9297091

[33] Khalil M, Teunissen CE, Lehmann S, et al Neurofilaments as biomarkers in neurological disorders—towards clinical application. Nat Rev Neurol. 2024;20(5):269–287. 38609644 10.1038/s41582-024-00955-x

[34] Rojas JC, Karydas A, Bang J, et al Plasma neurofilament light chain predicts progression in progressive supranuclear palsy. Ann Clin Transl Neurol. 2016;3(3):216–225. 27042681 10.1002/acn3.290 PMC4774256

[35] Kim KY, Shin KY, Chang KA. GFAP as a potential biomarker for Alzheimer’s disease: A systematic review and meta-analysis. Cells. 2023;12(9):1309. 37174709 10.3390/cells12091309 PMC10177296

[36] Quattrone A, Franzmeier N, Huppertz HJ, et al Magnetic resonance imaging measures to track atrophy progression in progressive supranuclear palsy in clinical trials. Mov Disord. 2024;39(8):1329–1342. 38825840 10.1002/mds.29866

[37] Street D, Bevan-Jones WR, Malpetti M, et al Structural correlates of survival in progressive supranuclear palsy. Parkinsonism Relat Disord. 2023;116:105866. 37804622 10.1016/j.parkreldis.2023.105866 PMC7615224

[38] Chiu WZ, Kaat LD, Seelaar H, et al Survival in progressive supranuclear palsy and frontotemporal dementia. J Neurol Neurosurg Psychiatry. 2010;81(4):441–445. 20360166 10.1136/jnnp.2009.195719

[39] Stern Y, Arenaza-Urquijo EM, Bartrés-Faz D, et al Whitepaper: Defining and investigating cognitive reserve, brain reserve, and brain maintenance. Alzheimer’s & Dementia. 2020;16(9):1305–1311. 10.1016/j.jalz.2018.07.219 PMC6417987 30222945

[40] Serra L, Cercignani M, Petrosini L, et al Neuroanatomical correlates of cognitive reserve in Alzheimer disease. Rejuvenation Res. 2011;14(2):143–151. 21204647 10.1089/rej.2010.1103

[41] Nutma E, Fancy N, Weinert M, et al Translocator protein is a marker of activated microglia in rodent models but not human neurodegenerative diseases. Nat Commun. 2023;14(1):5247. 37640701 10.1038/s41467-023-40937-z PMC10462763

[42] Kreisl WC, Kim MJ, Coughlin JM, Henter ID, Owen DR, Innis RB. PET imaging of neuroinflammation in neurological disorders. Lancet Neurol. 2020;19(11):940–950. 33098803 10.1016/S1474-4422(20)30346-X PMC7912433

[43] Nutma E, Stephenson JA, Gorter RP, et al A quantitative neuropathological assessment of translocator protein expression in multiple sclerosis. Brain. 2019;142(11):3440–3455. 31578541 10.1093/brain/awz287 PMC6821167

[44] Garland EF, Antony H, Kulagowska L, et al The microglial translocator protein (TSPO) in Alzheimer’s disease reflects a phagocytic phenotype. Acta Neuropathol. 2024;148(1):62. 39540994 10.1007/s00401-024-02822-x PMC11564344

[45] Turkheimer FE, Edison P, Pavese N, et al Reference and target region modeling of [11C]-(R)-PK11195 brain studies. J Nucl Med. 2007;48(1):158. 17204713

[46] Palleis C, Franzmeier N, Weidinger E, et al Association of neurofilament light chain, [18F]PI-2620 tau-PET, TSPO-PET, and clinical progression in patients with β-amyloid-negative CBS. Neurology. 2024;102(1):e207901. 38165362 10.1212/WNL.0000000000207901 PMC10834119

