# Supplementary material for: Inflammation PET and plasma neurofilament light predict survival in people with progressive supranuclear palsy
Source: Brain Commun. 2025 Nov 27;7(6):fcaf467. doi: 10.1093/braincomms/fcaf467 (PMC12709281; doi:10.1093/braincomms/fcaf467)
Supplement: fcaf467_Supplementary_Data [file fcaf467_supplementary_data.docx]

**Supplementary Material**

**Blood sample processing**

Plasma samples were thawed on wet ice, centrifuged at 500× g for 5 min at 4°C. Calibrators (neat) and samples plasma: 1:4 dilution) were measured in duplicates. The plasma assays used the Quanterix Simoa Human Neurology 4-Plex E assay (measuring Aβ40, Aβ42, GFAP and NfL ) and the Quanterix Simoa p-tau181 measuring p-tau181 of the human tau protein. Assays were performed using the Simoa-HD1 according to the manufacturer’s protocol (Quanterix Corp, Billerica, Massachusetts, USA) (Rissin et al). All samples were analysed at the same time using the same batch of reagents. A four-parameter logistic curve data reduction method was used to generate a calibration curve. Two control samples of known concentration of the protein of interest (high- control and low- control) were included as quality controls. All variables were log_10_-transformed prior to statistical analyses.

**Magnetic Resonance Imaging**

We visually inspected all 172 MRI scans to ensure high-quality data; then we resampled all scans to 1 mm^3^ isotropic resolution using the Advanced Normalization Tools (ANTS)^1^ version 2.3.5 function ResampleImage with a Gaussian Interpolation approach. Images were cropped to remove the neck using an in-house script running under MATLAB (MathWorks, inc. Natick, Massachusetts, USA) version 2020b. We segmented the scans into grey matter (GM), white matter (WM), cerebrospinal fluid (CSF), and estimated total intracranial volume (TIV=GM+WM+CSF) using the longitudinal analysis pipeline from the Computational Anatomy Toolbox 12 (CAT12) and statistical parametric mapping software (SPM12). We applied a modified version of the n30r83 Hammersmith atlas (www.brain-development.org), as previously used ^2^  ^3 4^ The modified Hammers atlas includes brainstem and cerebellar parcellations, as core regions for PSP, among 83 cortical and subcortical regions of interest (ROI) in every patient. As the scans were obtained with different MRI scanners and protocols, which might introduce noise and bias that could affect subsequent statistical analysis and interpretation, we applied a longitudinal harmonisation algorithm (Long Combat ^5^). All ROIs were then adjusted for TIV, and the left and right corresponding ROIs were summed, resulting in 43 bilateral regional volumes adjusted for TIV. Since both ROIs and TIV are measured in the same physical units (mL), the division yields a unitless ratio; the values were subsequently scaled by 1000 for numerical readability.

**Positron Emission Tomography**

For each participant with [^11^C]-PK11195 PET, the aligned dynamic PET image series for each scan was rigidly co-registered to the T1w MRI image. Non-displaceable binding potentials (BP_ND_) were calculated in the same 83 cortical and subcortical ROIs as for T1w MRI. Before kinetic modelling, regional PET data were corrected for partial volume effects from CSF by dividing by the mean regional GM plus WM fraction determined from SPM segmentation. For [^11^C]-PK11195, supervised cluster analysis was used to determine the reference tissue time-activity curve and BP_ND_ values were calculated in each ROI using a simplified reference tissue model with vascular binding correction ^6^. The CSF-partial-volume corrected BP_ND_ regional values were averaged across left and right hemisphere, resulting in the same 43 bilateral ROIs as in MRsI processing.

**Supplementary Figure 1.** Box plots of MRI visits across years in the MRI cohort. Red lines indicate patients who are also in the PET cohort.


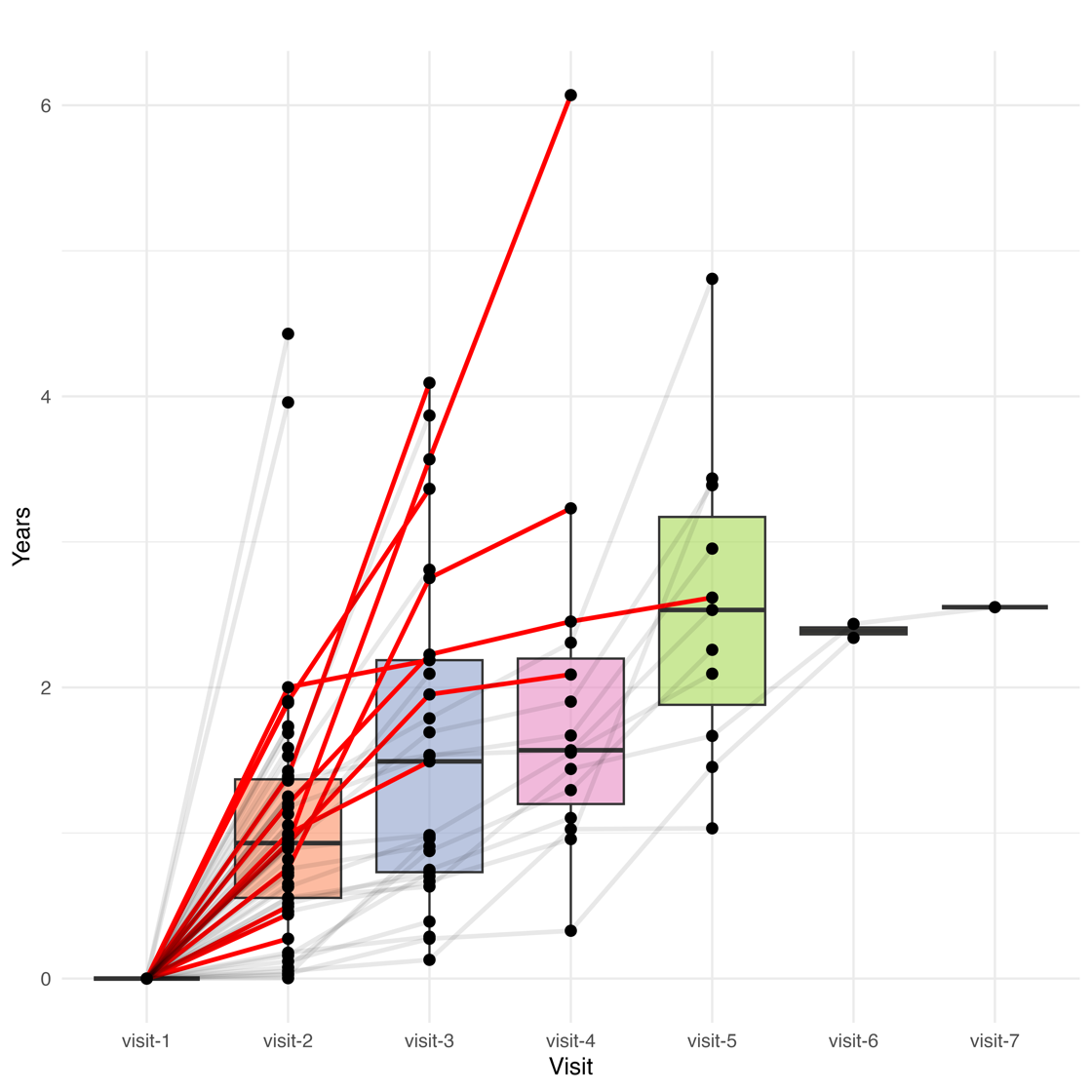


The number of participant visits at each time point was: Visit 1 (n = 59), Visit 2 (n = 55), Visit 3 (n = 29), Visit 4 (n = 15), Visit 5 (n = 11), Visit 6 (n = 2), and Visit 7 (n = 1). Each dot represents an individual participant, and the lines link individual follow-up visits for each participant. The y-axis represents years since baseline (Visit 1), indicating cumulative follow-up time.

**Supplementary Figure 2**. Distribution of survival times from MRI at Baseline (MRI Cohort) in years (bin). Red bars indicate participants who underwent both MRI and PET (PET cohort).


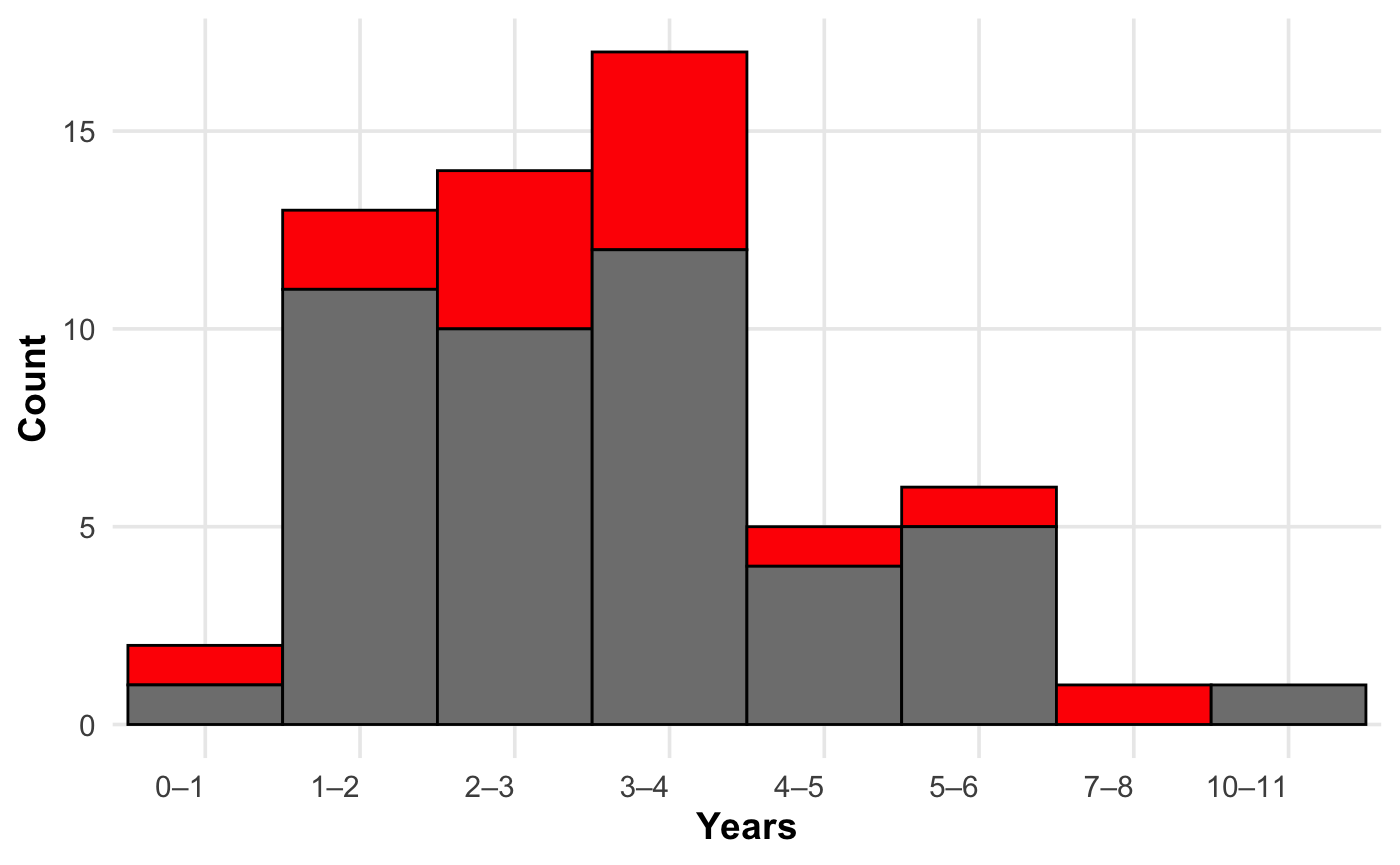


Participants per year bin: 0–1 (n=2), 1–2 (n=13), 2–3 (n=14), 3–4 (n=17), 4–5 (n=5), 5–6 (n=6), 7–8 (n=1), 10–11 (n=1).



**Supplementary Figure 3**. Kaplan-Meier Curves of the Biomarkers stratified by low, high, and medium for the MRI cohort, whereas for the PET cohort, biomarkers were stratified by low and high scores. For plot A, higher Component 2 MRI scores indicate more severe atrophy. In plot B, higher Component 2 MRI slope scores indicate a more rapid brain volume loss. For plot C, higher Progressive Supranuclear Palsy Rating Scale (PSPRS) scores indicate worse clinical severity. For plot D, higher Component 2 TSPO PET scores indicate higher neuroinflammation. For plot E, higher neurofilament light (NfL) levels indicate worse degeneration, and for plot F, higher Component 2 MRI Cross-sectional scores indicate more severe atrophy.


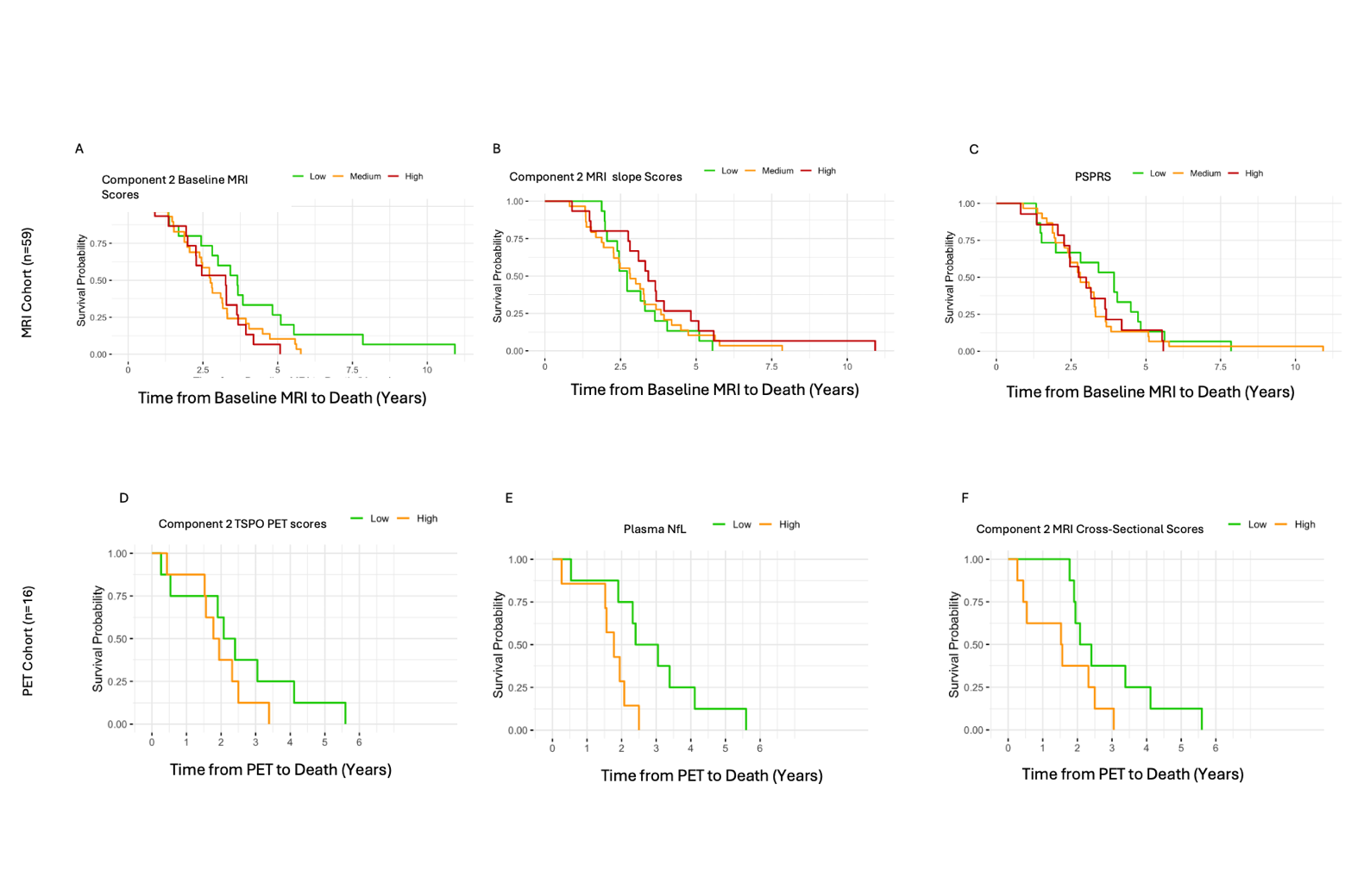


**Supplementary Figure 4**. Spaghetti plots for longitudinal trajectories of TIV-normalised bilateral regional volumes (ratios of volumes/TIV) across 43 regions of interest. Each line corresponds to one participant from baseline scan to death (n = 59).


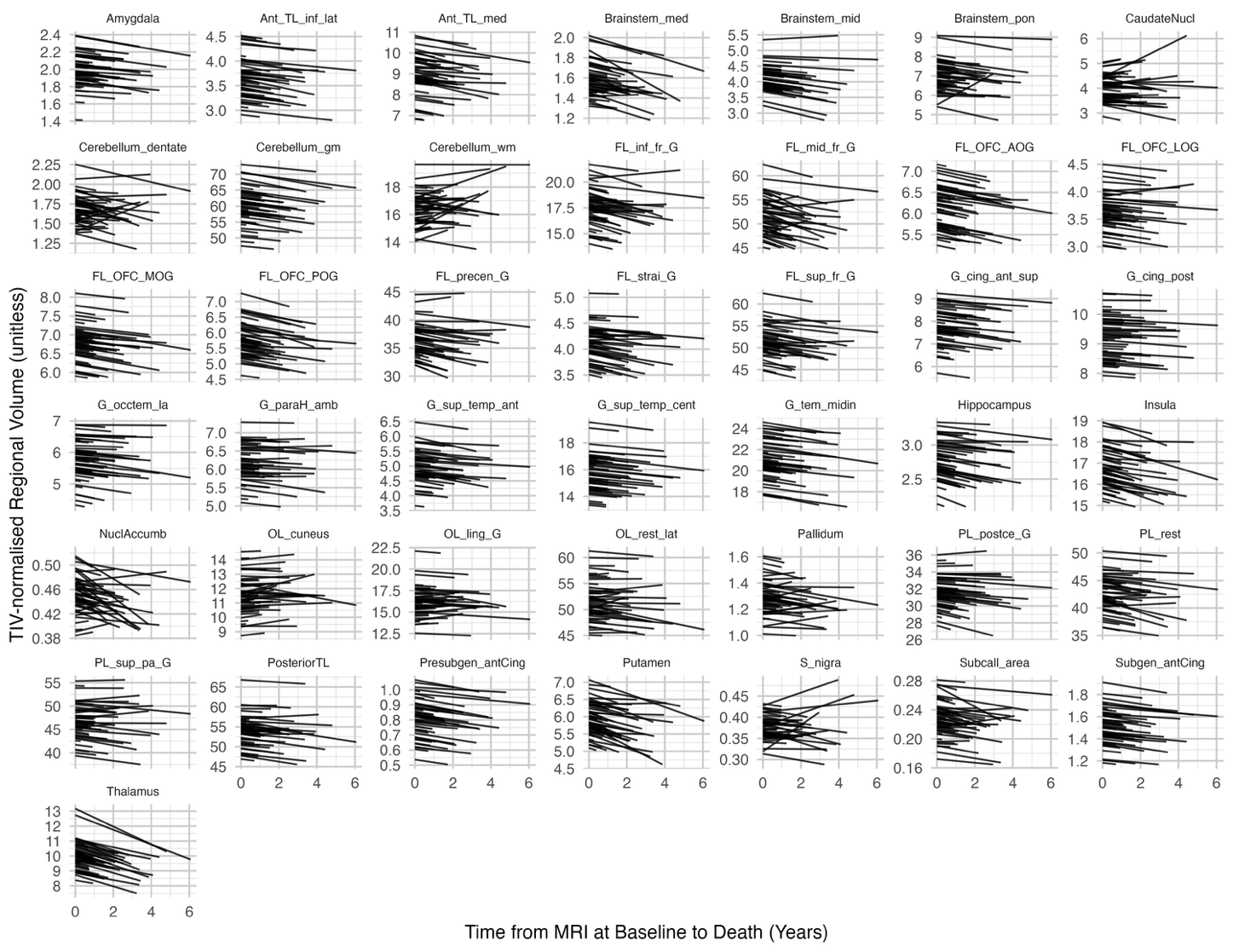


Anterior temporal lobe (medial part) = Ant_TL_med; Anterior temporal lobe (lateral part) = Ant_TL_inf_lat; Parahippocampal and ambient gyri = G_paraH_amb; Superior temporal gyrus (posterior part) = G_sup_temp_cent; Middle & inferior temporal gyrus = G_tem_midin; Fusiform gyrus = G_octem_la; Lateral remainder of occipital lobe = OL_rest_lat; Cingulate gyrus (anterior part) = G_cing_ant_sup; Cingulate gyrus (posterior part) = G_cing_post; Middle frontal gyrus = FL_mid_fr_G; Posterior temporal lobe = PosteriorTL; Inferolateral remainder of parietal lobe = PL_rest; Caudate nucleus = CaudateNucl; Nucleus accumbens = NuclAccumb; Precentral gyrus = FL_precen_G; Straight gyrus = FL_strai_G; Anterior orbital gyrus = FL_OFC_AOG; Inferior frontal gyrus = FL_inf_fr_G; Superior frontal gyrus = FL_sup_fr_G; Postcentral gyrus = PL_postce_G; Superior parietal gyrus = PL_sup_pa_G; Lingual gyrus = OL_ling_G; Cuneus = OL_cuneus; Medial orbital gyrus = FL_OFC_MOG; Lateral orbital gyrus = FL_OFC_LOG; Posterior orbital gyrus = FL_OFC_POG; Substantia nigra = S_nigra; Subgenual frontal cortex = Subgen_antCing; Subcallosal area = Subcall_area; Presubgenual frontal cortex = Presubgen_antCing; Superior temporal gyrus (anterior part) = G_sup_temp_ant; Cerebellum (GM) = Cerebellum_gm; Cerebellum (WM) = Cerebellum_wm; Cerebellum (dentate) = Cerebellum_dentate; Brainstem (medulla) = Brainstem_med; Brainstem (midbrain) = Brainstem_mid; Brainstem (pons) = Brainstem_pon.

**Supplementary Table 1**. An Analysis of Variance (ANOVA) Type 3 Satterthwaite’s method was performed on the resulting linear-fixed effect models to determine the effect of time on regional volume for each ROI.

| Regions | Sum Sq | Mean Sq | NumDF | DenDF | F value | Pr(>F) |
| --- | --- | --- | --- | --- | --- | --- |
| Brainstem_mid | 0.850 | 0.850 | 1 | 13.233 | 21.359 | 0.000 |
| Brainstem_pon | 0.473 | 0.473 | 1 | 33.095 | 4.179 | 0.049 |
| Brainstem_med | 0.098 | 0.098 | 1 | 39.745 | 15.972 | 0.000 |
| Hippocampus | 0.194 | 0.194 | 1 | 20.683 | 35.725 | 0.000 |
| Amygdala | 0.076 | 0.076 | 1 | 11.100 | 7.256 | 0.021 |
| Anterior_temporal_lobe_medial_part | 0.985 | 0.985 | 1 | 17.820 | 27.031 | 0.000 |
| Anterior_temporal_lobe_lateral_part | 0.243 | 0.243 | 1 | 18.797 | 18.907 | 0.000 |
| Parahippocampal_and_ambient_gyri | 0.085 | 0.085 | 1 | 15.046 | 2.354 | 0.146 |
| Superior_temporal_gyrus_posterior_part | 2.743 | 2.743 | 1 | 20.406 | 39.258 | 0.000 |
| Middle_and_inferior_temporal_gyrus | 6.129 | 6.129 | 1 | 13.251 | 33.681 | 0.000 |
| Fusiform_gyrus | 0.396 | 0.396 | 1 | 19.332 | 18.363 | 0.000 |
| Insula | 3.221 | 3.221 | 1 | 32.762 | 41.471 | 0.000 |
| Lateral_remainder_of_occipital_lobe | 1.316 | 1.316 | 1 | 17.243 | 1.389 | 0.255 |
| Cingulate_gyrus_anterior_part | 1.301 | 1.301 | 1 | 14.012 | 25.276 | 0.000 |
| Gyrus_cinguli_posterior_part | 0.159 | 0.159 | 1 | 15.552 | 2.817 | 0.113 |
| Middle_frontal_gyrus | 32.489 | 32.489 | 1 | 33.929 | 25.846 | 0.000 |
| Posterior_temporal_lobe | 9.798 | 9.798 | 1 | 33.753 | 14.401 | 0.001 |
| Inferiolateral_remainder_of_parietal_lobe | 9.361 | 9.361 | 1 | 40.356 | 21.056 | 0.000 |
| Caudate_nucleus | 0.001 | 0.001 | 1 | 38.455 | 0.026 | 0.873 |
| Nucleus_accumbens | 0.003 | 0.003 | 1 | 24.153 | 8.032 | 0.009 |
| Putamen | 1.488 | 1.488 | 1 | 28.071 | 23.750 | 0.000 |
| Thalamus | 13.459 | 13.459 | 1 | 30.217 | 58.281 | 0.000 |
| Pallidum | 0.009 | 0.009 | 1 | 19.578 | 2.317 | 0.144 |
| Precentral_gyrus | 13.426 | 13.426 | 1 | 24.618 | 27.564 | 0.000 |
| Straight_gyrus | 0.217 | 0.217 | 1 | 18.000 | 10.783 | 0.004 |
| Anterior_orbital_gyrus | 0.854 | 0.854 | 1 | 22.922 | 19.498 | 0.000 |
| Inferior_frontal_gyrus | 4.371 | 4.371 | 1 | 35.543 | 38.282 | 0.000 |
| Superior_frontal_gyrus | 33.154 | 33.154 | 1 | 20.487 | 23.056 | 0.000 |
| Postcentral_gyrus | 6.738 | 6.738 | 1 | 17.149 | 15.143 | 0.001 |
| Superior_parietal_gyrus | 4.688 | 4.688 | 1 | 28.542 | 5.437 | 0.027 |
| Lingual_gyrus | 0.031 | 0.031 | 1 | 30.351 | 0.135 | 0.715 |
| Cuneus | 0.240 | 0.240 | 1 | 32.469 | 1.806 | 0.188 |
| Medial_orbital_gyrus | 0.626 | 0.626 | 1 | 19.761 | 18.564 | 0.000 |
| Lateral_orbital_gyrus | 0.123 | 0.123 | 1 | 31.346 | 12.030 | 0.002 |
| Posterior_orbital_gyrus | 1.216 | 1.216 | 1 | 30.105 | 30.782 | 0.000 |
| Substantia_nigra | 0.000 | 0.000 | 1 | 35.502 | 0.039 | 0.845 |
| Subgenual_frontal_cortex | 0.046 | 0.046 | 1 | 10.831 | 20.125 | 0.001 |
| Subcallosal_area | 0.001 | 0.001 | 1 | 25.010 | 6.088 | 0.021 |
| Presubgenual_frontal_cortex | 0.037 | 0.037 | 1 | 18.396 | 24.541 | 0.000 |
| Superior_temporal_gyrus_anterior_part | 0.366 | 0.366 | 1 | 26.688 | 28.540 | 0.000 |
| Cerebellum_gm | 72.391 | 72.391 | 1 | 16.329 | 21.391 | 0.000 |
| Cerebellum_wm | 0.672 | 0.672 | 1 | 29.572 | 0.540 | 0.468 |
| Cerebellum_dentate | 0.034 | 0.034 | 1 | 36.300 | 2.708 | 0.108 |

**Supplementary Table 2**. Explorative partial Pearsons correlations (not adjusted for multiple comparisons) using the MRI cohort between MRI at baseline components and survival rate adjusted for disease duration

| Component | r | p-value |
| --- | --- | --- |
| Component 1 | -0.07 | 0.30 |
| Component 3 | -0.24 | 0.03 |
| Component 4 | -0.11 | 0.20 |
| Component 5 | -0.02 | 0.43 |
| Component 6 | -0.20 | 0.05 |
| Component 7 | -0.20 | 0.05 |
| Component 8 | 0.07 | 0.29 |
| Component 9 | 0.23 | 0.03 |
| Component 10 | 0.21 | 0.05 |

**Supplementary Table 3.** Explorative partial Pearsons correlations (not adjusted for multiple comparisons) using the MRI cohort between MRI-Slopes components and survival rate adjusted for disease duration

| Component | r | p-value |
| --- | --- | --- |
| Component 1 | -0.25 | 0.02 |
| Component 2 | 0.23 | 0.04 |
| Component 3 | 0.30 | 0.009 |
| Component 4 | 0.16 | 0.11 |
| Component 5 | 0.03 | 0.39 |
| Component 7 | 0.31 | 0.008 |
| Component 8 | -0.13 | 0.15 |
| Component 9 | 0.09 | 0.24 |
| Component 10 | -0.13 | 0.15 |

**Supplementary Table 4.** Explorative partial Spearman’s correlations (not adjusted for multiple comparisons) using the PET cohort between TSPO PET components and survival rate adjusted for disease duration

| Component | rho | p-value | Bayes Factor |
| --- | --- | --- | --- |
| Component 1 | 0.22 | 0.79 | 0.17 |
| Component 3 | 0.04 | 0.56 | 0.22 |
| Component 4 | -0.16 | 0.27 | 0.52 |

**Supplementary Table 5.** Explorative partial Spearman’s correlations (not adjusted for multiple comparisons) using the PET cohort between MRI-Cross Sectional components and survival rate adjusted for disease duration

| Component | rho | p-value | Bayes Factor |
| --- | --- | --- | --- |
| Component 1 | -0.07 | 0.39 | 0.50 |
| Component 3 | -0.38 | 0.06 | 1.46 |
| Component 4 | -0.02 | 0.49 | 0.29 |
| Component 5 | -0.04 | 0.44 | 0.47 |
| Component 6 | 0.07 | 0.61 | 0.03 |

**Supplementary Table 6.** Explorative partial Spearman’s correlations (not adjusted for multiple comparisons) using the PET cohort between plasma fluid biomarkers and survival adjusted for disease duration

| Biomarker | rho | p-value | Bayes Factor |
| --- | --- | --- | --- |
| GFAP | -0.16 | 0.28 | 0.49 |
| AB40 | -0.05 | 0.43 | 0.39 |
| AB42 | -0.22 | 0.22 | 0.56 |
| pTau181 | -0.22 | 0.22 | 0.70 |
| AB42 / AB40 | -0.26 | 0.19 | 0.75 |
| GFAP/NfL | 0.27 | 0.18 | 0.16 |

**Supplementary Table 7.** Regional rotated loading of the four [^11^C]-PK11195 PET components derived by principal component analysis on non-displaceable binding potential regional values (PET Cohort).

| Regions | Dim.1 | Dim.2 | Dim.3 | Dim.4 |
| --- | --- | --- | --- | --- |
| Hippocampus | 0.185 | 0.342 | -0.713 | -0.231 |
| Amygdala | 0.178 | 0.483 | -0.741 | -0.119 |
| Anterior_temporal_lobe_medial_part | 0.313 | 0.083 | -0.911 | 0.016 |
| Anterior_temporal_lobe_lateral_part | 0.621 | 0.151 | -0.704 | -0.133 |
| Parahippocampal_and_ambient_gyri | 0.500 | 0.211 | -0.714 | -0.138 |
| Superior_temporal_gyrus_posterior_part | 0.729 | 0.294 | -0.503 | -0.151 |
| Middle_and_inferior_temporal_gyrus | 0.727 | 0.248 | -0.599 | -0.047 |
| Fusiform_gyrus | 0.575 | 0.241 | -0.513 | 0.010 |
| Insula | 0.540 | 0.400 | -0.672 | -0.159 |
| Lateral_remainder_of_occipital_lobe | 0.674 | 0.261 | -0.596 | -0.216 |
| Cingulate_gyrus_anterior_part | 0.531 | 0.006 | -0.666 | 0.022 |
| Gyrus_cinguli_posterior_part | 0.702 | 0.166 | -0.596 | -0.029 |
| Middle_frontal_gyrus | 0.626 | 0.279 | -0.173 | -0.326 |
| Posterior_temporal_lobe | 0.699 | 0.297 | -0.629 | -0.102 |
| Inferiolateral_remainder_of_parietal_lobe | 0.660 | 0.144 | -0.668 | -0.160 |
| Caudate_nucleus | 0.681 | 0.162 | -0.349 | 0.458 |
| Nucleus_accumbens | 0.406 | -0.099 | -0.509 | 0.466 |
| Putamen | 0.540 | 0.355 | -0.253 | 0.159 |
| Thalamus | 0.857 | 0.266 | -0.203 | -0.014 |
| Pallidum | 0.012 | 0.732 | -0.149 | 0.118 |
| Precentral_gyrus | 0.393 | 0.443 | -0.372 | -0.592 |
| Straight_gyrus | 0.759 | 0.087 | -0.564 | 0.168 |
| Anterior_orbital_gyrus | 0.844 | 0.267 | -0.328 | 0.058 |
| Inferior_frontal_gyrus | 0.829 | 0.146 | -0.451 | -0.090 |
| Superior_frontal_gyrus | 0.721 | 0.095 | -0.039 | -0.234 |
| Postcentral_gyrus | 0.547 | 0.161 | -0.583 | -0.332 |
| Superior_parietal_gyrus | 0.562 | 0.046 | -0.677 | -0.343 |
| Lingual_gyrus | 0.799 | 0.140 | -0.497 | 0.017 |
| Cuneus | 0.802 | 0.123 | -0.439 | -0.022 |
| Medial_orbital_gyrus | 0.847 | 0.211 | -0.409 | 0.096 |
| Lateral_orbital_gyrus | 0.795 | 0.050 | -0.505 | -0.037 |
| Posterior_orbital_gyrus | 0.644 | 0.271 | -0.667 | -0.054 |
| Substantia_nigra | -0.047 | 0.028 | -0.110 | -0.715 |
| Subgenual_frontal_cortex | 0.691 | 0.349 | -0.456 | 0.215 |
| Subcallosal_area | 0.348 | 0.096 | -0.732 | 0.505 |
| Presubgenual_frontal_cortex | 0.841 | 0.142 | -0.293 | 0.098 |
| Superior_temporal_gyrus_anterior_part | 0.538 | 0.068 | -0.781 | 0.216 |
| Cerebellum_gm | 0.850 | 0.019 | -0.320 | 0.239 |
| Cerebellum_wm | 0.093 | 0.798 | -0.352 | -0.116 |
| Cerebellum_dentate | 0.221 | 0.813 | 0.044 | -0.058 |
| Brainstem_med | 0.177 | 0.495 | -0.504 | -0.440 |
| Brainstem_mid | 0.645 | 0.526 | -0.175 | -0.335 |
| Brainstem_pon | 0.355 | 0.829 | -0.057 | -0.123 |
| **Cumulative % of var.** | **64** | **72** | **78** | **82** |

**Supplementary Table 8.** Regional rotated loading of the 6 components derived by principal component analysis on MRI Cross Sectional (PET Cohort).

| Regions | Dim.1 | Dim.2 | Dim.3 | Dim.4 | Dim.5 | Dim.6 |
| --- | --- | --- | --- | --- | --- | --- |
| Brainstem_mid | -0.730 | -0.598 | 0.181 | -0.034 | -0.056 | -0.050 |
| Brainstem_pon | -0.458 | -0.827 | -0.090 | 0.052 | 0.107 | -0.061 |
| Brainstem_med | -0.241 | -0.833 | -0.125 | -0.133 | -0.041 | -0.273 |
| Hippocampus | -0.179 | -0.559 | -0.702 | -0.019 | 0.088 | 0.092 |
| Amygdala | 0.122 | -0.521 | -0.692 | 0.057 | 0.004 | 0.086 |
| Anterior_temporal_lobe_medial_part | 0.521 | -0.250 | -0.718 | 0.086 | -0.135 | -0.029 |
| Anterior_temporal_lobe_lateral_part | 0.445 | -0.533 | -0.375 | -0.148 | 0.019 | -0.264 |
| Parahippocampal_and_ambient_gyri | 0.214 | -0.257 | -0.419 | 0.406 | 0.164 | 0.269 |
| Superior_temporal_gyrus_posterior_part | -0.291 | 0.039 | -0.785 | -0.155 | 0.132 | -0.187 |
| Middle_and_inferior_temporal_gyrus | -0.106 | 0.099 | -0.726 | -0.284 | 0.036 | -0.309 |
| Fusiform_gyrus | 0.274 | 0.417 | -0.688 | -0.214 | 0.248 | -0.149 |
| Insula | -0.723 | 0.231 | -0.352 | -0.158 | -0.021 | 0.072 |
| Lateral_remainder_of_occipital_lobe | -0.673 | 0.532 | 0.061 | -0.060 | -0.184 | -0.181 |
| Cingulate_gyrus_anterior_part | -0.751 | -0.003 | -0.218 | 0.307 | 0.259 | 0.018 |
| Gyrus_cinguli_posterior_part | -0.801 | 0.391 | 0.159 | 0.052 | 0.203 | -0.038 |
| Middle_frontal_gyrus | -0.911 | 0.071 | -0.287 | 0.162 | 0.038 | -0.084 |
| Posterior_temporal_lobe | -0.498 | 0.380 | -0.471 | -0.293 | 0.252 | -0.240 |
| Inferiolateral_remainder_of_parietal_lobe | -0.687 | 0.332 | -0.082 | 0.025 | 0.407 | -0.148 |
| Caudate_nucleus | -0.784 | 0.050 | -0.001 | 0.040 | -0.435 | -0.051 |
| Nucleus_accumbens | -0.478 | -0.303 | -0.015 | -0.605 | -0.401 | -0.108 |
| Putamen | -0.819 | -0.278 | -0.294 | -0.085 | -0.039 | 0.072 |
| Thalamus | -0.569 | -0.609 | 0.154 | 0.132 | -0.116 | -0.050 |
| Pallidum | -0.760 | -0.238 | 0.134 | -0.264 | -0.434 | -0.160 |
| Precentral_gyrus | -0.692 | 0.104 | -0.280 | 0.496 | 0.255 | -0.092 |
| Straight_gyrus | -0.222 | 0.516 | -0.134 | -0.469 | -0.107 | 0.267 |
| Anterior_orbital_gyrus | -0.576 | 0.098 | -0.208 | 0.352 | -0.371 | 0.291 |
| Inferior_frontal_gyrus | -0.787 | 0.082 | 0.019 | -0.195 | 0.174 | 0.456 |
| Superior_frontal_gyrus | -0.774 | 0.290 | -0.232 | 0.373 | -0.028 | -0.047 |
| Postcentral_gyrus | -0.619 | 0.242 | -0.064 | 0.598 | 0.014 | 0.142 |
| Superior_parietal_gyrus | -0.800 | 0.244 | 0.237 | 0.246 | 0.185 | 0.095 |
| Lingual_gyrus | -0.360 | 0.267 | 0.073 | 0.051 | -0.677 | -0.342 |
| Cuneus | -0.381 | 0.417 | -0.171 | 0.305 | -0.233 | -0.552 |
| Medial_orbital_gyrus | -0.735 | 0.373 | -0.226 | 0.120 | -0.180 | 0.267 |
| Lateral_orbital_gyrus | -0.381 | -0.126 | -0.169 | -0.044 | -0.537 | 0.313 |
| Posterior_orbital_gyrus | -0.149 | 0.154 | -0.513 | -0.357 | -0.174 | 0.612 |
| Substantia_nigra | -0.759 | -0.371 | 0.232 | 0.114 | -0.004 | -0.118 |
| Subgenual_frontal_cortex | -0.676 | 0.181 | 0.081 | -0.633 | 0.193 | -0.033 |
| Subcallosal_area | -0.384 | -0.127 | 0.234 | -0.563 | -0.019 | 0.087 |
| Presubgenual_frontal_cortex | -0.567 | -0.038 | 0.107 | -0.527 | 0.463 | 0.018 |
| Superior_temporal_gyrus_anterior_part | 0.581 | -0.206 | -0.607 | 0.174 | -0.268 | 0.109 |
| Cerebellum_gm | -0.293 | -0.806 | 0.028 | 0.034 | 0.019 | 0.013 |
| Cerebellum_wm | -0.545 | -0.717 | 0.215 | 0.027 | 0.166 | 0.139 |
| Cerebellum_dentate | -0.608 | -0.623 | 0.285 | 0.128 | 0.230 | -0.011 |
| **Cumulative % of var.** | **33** | **50** | **62** | **70** | **76** | **81** |

**Supplementary Table 9.** Regional rotated loading of the 10 components derived by principal component analysis on MRI-Slopes (MRI Cohort).

| Regions | Dim.1 | Dim.2 | Dim.3 | Dim.4 | Dim.5 | Dim.6 | Dim.7 | Dim.8 | Dim.9 | Dim.10 |
| --- | --- | --- | --- | --- | --- | --- | --- | --- | --- | --- |
| Brainstem_mid | 0.164 | -0.225 | -0.171 | 0.095 | -0.103 | -0.084 | -0.500 | 0.516 | -0.024 | 0.158 |
| Brainstem_pon | 0.068 | 0.153 | -0.111 | 0.114 | 0.086 | 0.840 | -0.194 | 0.092 | -0.141 | 0.077 |
| Brainstem_med | -0.183 | -0.156 | 0.100 | 0.149 | 0.010 | 0.804 | 0.291 | -0.014 | -0.137 | 0.123 |
| Hippocampus | 0.166 | -0.185 | -0.205 | -0.680 | -0.076 | -0.138 | -0.224 | 0.049 | -0.101 | 0.068 |
| Amygdala | 0.171 | 0.177 | -0.007 | -0.049 | -0.262 | 0.529 | 0.253 | 0.178 | 0.373 | 0.235 |
| Anterior_temporal_lobe_medial_part | 0.272 | 0.146 | 0.021 | -0.538 | -0.367 | 0.315 | -0.083 | 0.327 | 0.072 | 0.002 |
| Anterior_temporal_lobe_lateral_part | 0.292 | -0.026 | -0.055 | -0.326 | -0.682 | 0.066 | 0.112 | 0.253 | 0.273 | 0.217 |
| Parahippocampal_and_ambient_gyri | -0.060 | 0.019 | -0.018 | -0.717 | -0.023 | -0.131 | -0.005 | -0.144 | -0.346 | -0.132 |
| Superior_temporal_gyrus_posterior_part | 0.277 | 0.013 | 0.316 | -0.290 | -0.142 | 0.266 | 0.161 | 0.451 | -0.250 | 0.012 |
| Middle_and_inferior_temporal_gyrus | 0.225 | -0.106 | 0.314 | -0.676 | -0.285 | -0.123 | 0.101 | 0.215 | -0.059 | -0.003 |
| Fusiform_gyrus | 0.158 | 0.013 | 0.116 | -0.799 | 0.060 | 0.016 | 0.079 | -0.138 | 0.078 | 0.060 |
| Insula | 0.637 | -0.064 | 0.167 | -0.418 | -0.128 | 0.218 | 0.016 | 0.422 | -0.193 | -0.012 |
| Lateral_remainder_of_occipital_lobe | 0.086 | -0.107 | 0.852 | -0.123 | -0.101 | -0.104 | 0.014 | -0.128 | -0.077 | 0.019 |
| Cingulate_gyrus_anterior_part | 0.755 | -0.420 | -0.079 | -0.076 | -0.216 | 0.068 | -0.146 | 0.009 | 0.081 | 0.088 |
| Gyrus_cinguli_posterior_part | 0.123 | -0.854 | 0.091 | 0.027 | -0.103 | 0.101 | -0.060 | -0.078 | 0.071 | 0.107 |
| Middle_frontal_gyrus | 0.869 | -0.191 | 0.208 | 0.128 | -0.172 | -0.026 | 0.062 | 0.054 | -0.004 | 0.008 |
| Posterior_temporal_lobe | 0.116 | -0.294 | 0.571 | -0.509 | -0.290 | -0.100 | 0.092 | 0.012 | -0.167 | 0.011 |
| Inferiolateral_remainder_of_parietal_lobe | 0.402 | -0.603 | 0.506 | -0.143 | -0.088 | 0.001 | 0.020 | -0.007 | -0.211 | 0.038 |
| Caudate_nucleus | 0.170 | 0.024 | -0.089 | 0.223 | -0.115 | -0.012 | 0.033 | 0.773 | -0.020 | -0.026 |
| Nucleus_accumbens | 0.181 | 0.214 | 0.239 | -0.045 | -0.195 | 0.116 | -0.266 | 0.096 | 0.398 | 0.586 |
| Putamen | 0.280 | -0.055 | -0.096 | -0.305 | -0.101 | 0.283 | -0.059 | 0.679 | 0.181 | 0.199 |
| Thalamus | -0.095 | 0.121 | 0.184 | 0.085 | -0.315 | 0.116 | 0.778 | 0.085 | 0.043 | 0.180 |
| Pallidum | -0.010 | 0.268 | 0.162 | 0.111 | 0.106 | 0.372 | -0.011 | 0.123 | -0.681 | -0.326 |
| Precentral_gyrus | 0.450 | -0.659 | 0.025 | -0.033 | 0.102 | -0.018 | -0.150 | 0.143 | 0.143 | -0.265 |
| Straight_gyrus | 0.479 | -0.282 | -0.146 | -0.297 | 0.214 | -0.085 | 0.470 | 0.038 | 0.033 | -0.056 |
| Anterior_orbital_gyrus | 0.857 | -0.160 | 0.008 | -0.293 | -0.076 | 0.069 | -0.088 | 0.006 | -0.039 | -0.047 |
| Inferior_frontal_gyrus | 0.837 | -0.211 | -0.100 | -0.064 | -0.193 | 0.068 | 0.057 | 0.191 | 0.200 | -0.017 |
| Superior_frontal_gyrus | 0.868 | -0.293 | 0.107 | -0.022 | -0.136 | 0.022 | -0.009 | 0.066 | 0.127 | 0.017 |
| Postcentral_gyrus | 0.157 | -0.677 | -0.059 | -0.186 | 0.102 | -0.171 | 0.029 | 0.171 | 0.102 | -0.127 |
| Superior_parietal_gyrus | 0.311 | -0.776 | 0.367 | -0.035 | 0.006 | -0.135 | 0.024 | -0.205 | -0.013 | -0.103 |
| Lingual_gyrus | -0.258 | -0.041 | 0.695 | 0.185 | -0.114 | 0.195 | 0.102 | 0.012 | -0.255 | 0.230 |
| Cuneus | 0.047 | -0.059 | 0.922 | -0.008 | 0.233 | -0.002 | 0.078 | -0.015 | -0.021 | -0.010 |
| Medial_orbital_gyrus | 0.662 | -0.247 | -0.044 | -0.421 | 0.325 | -0.113 | 0.276 | 0.018 | 0.071 | -0.081 |
| Lateral_orbital_gyrus | 0.776 | -0.296 | 0.016 | -0.327 | 0.019 | -0.181 | -0.071 | 0.029 | 0.036 | 0.077 |
| Posterior_orbital_gyrus | 0.701 | 0.320 | -0.104 | -0.114 | -0.223 | 0.014 | -0.180 | 0.237 | 0.118 | -0.168 |
| Substantia_nigra | 0.216 | 0.398 | -0.221 | -0.145 | 0.081 | 0.447 | -0.442 | 0.059 | -0.365 | 0.140 |
| Subgenual_frontal_cortex | 0.621 | 0.243 | 0.093 | 0.089 | 0.075 | 0.048 | 0.342 | 0.465 | 0.095 | 0.142 |
| Subcallosal_area | 0.260 | 0.255 | -0.083 | -0.145 | 0.073 | -0.001 | 0.266 | 0.389 | -0.019 | 0.577 |
| Presubgenual_frontal_cortex | 0.828 | 0.134 | 0.000 | -0.053 | 0.019 | -0.027 | -0.124 | 0.335 | 0.135 | 0.148 |
| Superior_temporal_gyrus_anterior_part | 0.454 | -0.038 | 0.061 | -0.122 | -0.752 | -0.126 | 0.142 | 0.121 | 0.107 | 0.163 |
| Cerebellum_gm | -0.184 | -0.045 | 0.137 | 0.106 | -0.253 | 0.345 | 0.076 | 0.004 | -0.095 | 0.725 |
| Cerebellum_wm | -0.147 | 0.096 | 0.160 | -0.148 | 0.002 | 0.054 | -0.100 | -0.179 | -0.842 | 0.203 |
| Cerebellum_dentate | -0.178 | 0.001 | 0.144 | -0.182 | 0.111 | 0.044 | 0.009 | 0.100 | -0.833 | -0.046 |
| **Cumulative % of var.** | **26** | **38** | **48** | **56** | **63** | **68** | **72** | **75** | **78** | **80** |

**Supplementary Table 10.** Regional rotated loading of the 10 components derived by principal component analysis on MRI at Baseline (MRI Cohort).

| Regions | Dim.1 | Dim.2 | Dim.3 | Dim.4 | Dim.5 | Dim.6 | Dim.7 | Dim.8 | Dim.9 | Dim.10 |
| --- | --- | --- | --- | --- | --- | --- | --- | --- | --- | --- |
| Brainstem_mid | -0.064 | -0.804 | 0.176 | 0.063 | 0.096 | 0.085 | -0.170 | -0.176 | -0.099 | 0.152 |
| Brainstem_pon | -0.208 | -0.826 | -0.161 | -0.022 | 0.152 | -0.105 | -0.012 | -0.177 | -0.021 | -0.070 |
| Brainstem_med | -0.040 | -0.740 | -0.216 | -0.077 | 0.202 | -0.082 | -0.079 | -0.290 | -0.003 | 0.231 |
| Hippocampus | -0.175 | -0.247 | -0.670 | -0.178 | 0.339 | -0.088 | -0.182 | 0.073 | -0.135 | 0.316 |
| Amygdala | -0.012 | -0.222 | -0.682 | -0.151 | 0.451 | -0.225 | -0.091 | 0.059 | -0.007 | 0.041 |
| Anterior_temporal_lobe_medial_part | -0.022 | -0.017 | -0.931 | -0.052 | 0.008 | -0.028 | -0.131 | -0.061 | 0.039 | -0.009 |
| Anterior_temporal_lobe_lateral_part | 0.069 | -0.103 | -0.534 | -0.151 | 0.101 | -0.029 | -0.231 | -0.584 | 0.150 | 0.025 |
| Parahippocampal_and_ambient_gyri | -0.108 | -0.223 | -0.634 | -0.284 | 0.081 | 0.179 | -0.139 | 0.138 | -0.242 | 0.129 |
| Superior_temporal_gyrus_posterior_part | -0.450 | -0.060 | -0.505 | 0.017 | -0.180 | -0.437 | 0.009 | -0.224 | -0.121 | -0.008 |
| Middle_and_inferior_temporal_gyrus | -0.338 | 0.005 | -0.600 | -0.165 | 0.086 | -0.283 | -0.159 | -0.349 | -0.232 | -0.127 |
| Fusiform_gyrus | -0.373 | 0.152 | -0.695 | 0.042 | -0.237 | -0.064 | 0.174 | 0.022 | -0.210 | -0.013 |
| Insula | -0.503 | -0.163 | -0.191 | -0.156 | 0.377 | -0.221 | -0.062 | -0.005 | -0.435 | 0.235 |
| Lateral_remainder_of_occipital_lobe | -0.351 | -0.042 | -0.129 | -0.714 | 0.179 | -0.231 | -0.247 | 0.012 | -0.263 | 0.019 |
| Cingulate_gyrus_anterior_part | -0.595 | -0.131 | -0.208 | 0.028 | 0.162 | -0.351 | -0.322 | 0.361 | -0.032 | 0.009 |
| Gyrus_cinguli_posterior_part | -0.747 | -0.077 | 0.090 | -0.160 | 0.061 | -0.207 | -0.276 | 0.176 | -0.124 | 0.058 |
| Middle_frontal_gyrus | -0.764 | -0.098 | -0.097 | -0.104 | 0.264 | -0.195 | -0.343 | -0.055 | 0.001 | 0.148 |
| Posterior_temporal_lobe | -0.610 | -0.068 | -0.393 | -0.164 | 0.016 | -0.396 | 0.036 | -0.062 | -0.371 | 0.015 |
| Inferiolateral_remainder_of_parietal_lobe | -0.793 | -0.093 | -0.194 | -0.027 | 0.012 | -0.312 | 0.013 | -0.182 | -0.174 | 0.186 |
| Caudate_nucleus | -0.108 | -0.182 | 0.080 | -0.155 | 0.775 | 0.196 | 0.047 | -0.002 | -0.175 | 0.054 |
| Nucleus_accumbens | -0.038 | 0.035 | -0.091 | -0.264 | 0.681 | -0.369 | -0.141 | -0.142 | 0.091 | 0.088 |
| Putamen | -0.200 | -0.290 | -0.036 | -0.036 | 0.813 | -0.270 | -0.031 | 0.048 | -0.066 | 0.049 |
| Thalamus | -0.258 | -0.335 | -0.129 | 0.022 | 0.180 | -0.114 | -0.072 | 0.006 | -0.022 | 0.797 |
| Pallidum | -0.139 | -0.494 | 0.060 | -0.249 | 0.248 | 0.060 | -0.011 | -0.058 | -0.614 | 0.152 |
| Precentral_gyrus | -0.776 | -0.415 | -0.052 | -0.081 | 0.161 | 0.090 | -0.062 | 0.031 | -0.012 | 0.074 |
| Straight_gyrus | -0.206 | 0.107 | -0.141 | -0.052 | 0.011 | -0.272 | -0.407 | 0.111 | -0.677 | -0.133 |
| Anterior_orbital_gyrus | -0.396 | 0.033 | -0.152 | -0.321 | 0.128 | 0.020 | -0.649 | -0.068 | -0.042 | 0.097 |
| Inferior_frontal_gyrus | -0.480 | -0.121 | -0.057 | -0.087 | 0.122 | -0.212 | -0.413 | -0.052 | -0.254 | 0.357 |
| Superior_frontal_gyrus | -0.777 | -0.063 | -0.059 | -0.047 | -0.036 | -0.146 | -0.274 | 0.065 | -0.053 | -0.025 |
| Postcentral_gyrus | -0.779 | -0.260 | -0.093 | -0.022 | 0.167 | 0.167 | -0.099 | -0.033 | -0.026 | -0.156 |
| Superior_parietal_gyrus | -0.679 | -0.113 | 0.120 | -0.384 | -0.105 | -0.100 | -0.204 | -0.049 | -0.031 | 0.197 |
| Lingual_gyrus | -0.054 | -0.202 | -0.156 | -0.821 | 0.105 | -0.150 | -0.061 | 0.025 | -0.141 | -0.045 |
| Cuneus | -0.139 | -0.019 | -0.183 | -0.841 | 0.165 | 0.018 | -0.124 | -0.094 | 0.060 | 0.012 |
| Medial_orbital_gyrus | -0.403 | 0.071 | -0.118 | -0.223 | 0.068 | -0.080 | -0.575 | 0.076 | -0.433 | 0.127 |
| Lateral_orbital_gyrus | -0.371 | 0.005 | -0.300 | -0.077 | 0.172 | -0.041 | -0.683 | -0.062 | -0.066 | 0.172 |
| Posterior_orbital_gyrus | -0.214 | -0.029 | -0.168 | -0.074 | -0.149 | -0.203 | -0.765 | -0.081 | -0.045 | -0.127 |
| Substantia_nigra | -0.247 | -0.767 | 0.143 | -0.196 | 0.117 | -0.041 | -0.100 | -0.139 | -0.146 | -0.375 |
| Subgenual_frontal_cortex | -0.439 | -0.130 | -0.004 | -0.178 | 0.162 | -0.644 | -0.373 | 0.010 | -0.226 | -0.036 |
| Subcallosal_area | -0.138 | -0.189 | -0.094 | -0.234 | 0.191 | -0.668 | -0.003 | -0.073 | -0.023 | 0.249 |
| Presubgenual_frontal_cortex | -0.374 | -0.198 | -0.065 | 0.027 | 0.108 | -0.663 | -0.328 | 0.176 | -0.106 | -0.054 |
| Superior_temporal_gyrus_anterior_part | 0.087 | -0.084 | -0.782 | -0.059 | -0.104 | -0.009 | -0.172 | -0.032 | 0.121 | 0.038 |
| Cerebellum_gm | 0.016 | -0.723 | -0.253 | -0.123 | -0.001 | -0.280 | 0.003 | 0.333 | 0.067 | 0.172 |
| Cerebellum_wm | -0.126 | -0.851 | -0.115 | -0.132 | 0.025 | -0.131 | 0.138 | 0.287 | 0.038 | 0.057 |
| Cerebellum_dentate | -0.246 | -0.806 | -0.141 | 0.022 | 0.026 | -0.069 | 0.127 | 0.199 | 0.001 | 0.150 |
| **Cumulative % of var.** | **34** | **45** | **54** | **60** | **64** | **68** | **72** | **76** | **77** | **80** |

**Supplementary Table 11.** MRI manufacturers by models and scanning parameters.

| Manufacturer | Siemens | General Electric |
| --- | --- | --- |
| Model | TrioTim  Verio  Skyra  Prisma | Signa* |
| Slice and thickness | 256 of 1.00 mm thickness  256 of 1.10 mm thickness  256 of 1.20 mm thickness  192 of 1.25 mm thickness | 512 of 1.00 mm thickness |
| Echo time (s) | 0.00286  0.00298  0.00293  0.00288  0.00287  0.00285  0.00292 | 0.003432 0.003604 |
| Repetition Time (s) | 2.3  2.0 | 0.008876  0.0092 |
| Acquisition matrix | 144 x 192  176 x 240  208 x 256 | 192x512 |
| Voxel size (mm3) | 1.25 x 1.25 x 1.25  1 x 1 x 1   - 1. x 1.1 x 1.1   2. x 1 x 1 | 1 x 0.5469 x 0.5469 |

**References**

1. Avants BB, Tustison NJ, Song G, Cook PA, Klein A, Gee JC. A reproducible evaluation of ANTs similarity metric performance in brain image registration. *NeuroImage*. 2011;54(3):2033-2044. doi:10.1016/j.neuroimage.2010.09.025

2. Passamonti L, Rodríguez PV, Hong YT, et al. [11C]PK11195 binding in Alzheimer disease and progressive supranuclear palsy. *Neurology*. 2018;90(22):1989-1996. doi:10.1212/wnl.0000000000005610

3. Malpetti M, Passamonti L, Rittman T, et al. Neuroinflammation and Tau Colocalize in vivo in Progressive Supranuclear Palsy. *Annals of Neurology*. 2020;88(6):1194-1204. doi:10.1002/ana.25911

4. Malpetti M, Passamonti L, Jones PS, et al. Neuroinflammation predicts disease progression in progressive supranuclear palsy. *J Neurol Neurosurg Psychiatry*. 2021;92(7):769-775. doi:10.1136/jnnp-2020-325549

5. Beer JC, Tustison NJ, Cook PA, et al. Longitudinal ComBat: A method for harmonizing longitudinal multi-scanner imaging data. *NeuroImage*. 2020;220:117129. doi:10.1016/j.neuroimage.2020.117129

6. Yaqub M, Van Berckel BNM, Schuitemaker A, et al. Optimization of supervised cluster analysis for extracting reference tissue input curves in (R)-[ 11C]PK11195 brain PET studies. *Journal of Cerebral Blood Flow and Metabolism*. 2012;32(8):1600-1608. doi:10.1038/jcbfm.2012.59
